# Genetic diversity in nutritional composition of oat (*Avena sativa* L.) germplasm reported from Pakistan

**DOI:** 10.1016/j.sjbs.2021.11.023

**Published:** 2021-11-19

**Authors:** Mohammad Ihsan, Mohammad Nisar, Nausheen Nazir, Muhammad Zahoor, Atif Ali Khan Khalil, Abdul Ghafoor, Arshad Khan, Ramzi A. Mothana, Riaz Ullah, Nisar Ahmad

**Affiliations:** a Department of Botany, University of Malakand, Chakdara, Dir (Lower) – 18800, Khyber Pakhtun khwa, Pakistan; b Department of Bio-Chemistry, University of Malakand, Chakdara, Dir (Lower) – 18800, Khyber Pakhtun khwa, Pakistan; c Department of Biological Sciences, National University of Medical Sciences, 46000 Rawalpindi, Pakistan; d Pakistan Agriculture Research Council, Islamabad, Pakistan; e Department of Pharmacognosy, College of Pharmacy King Saud University, Riyadh, Saudi Arabia; f Center for Organismal Studies, Department of Molecular Biology of Plants, Im Neuenheimer Feld 360, 69120, University of Heildelberg, Germany

**Keywords:** Oat, Minerals composition, Elemental profile, Antioxidants, HPLC-finger printing, PCA, Principal Component analysis, CA, Cluster analysis, C.V, Cumulative Variance, CHO, Carbohydrates, AOAC, Association of Official Analytical Chemists, Ml, Milliliter, HPLC-UV, High performance liquid chromatography- ultraviolet, Met. Ext, Methanolic extract, Mg, milligrams, µg, Microgram, DPPH, 2, 2-Diphenyl, 1, picrylhydrazyl, ABTS, 2, 2′-Azino-bis (3-ethylbenzothiazoline-6-sulfonic acid, IC _50_, Minimum inhibitory concentration

## Abstract

In the present study, 30 potential germplasm of oat (*Avena sativa* L.) were subjected to proximate, elemental, and HPLC analysis to provide a scientific basis to genetic diversity present among them. The extracts of the selected germplasms were also evaluated for their antioxidant potentials through DPPH and ABTS assays. Proximate analysis showed protein contents to be in the range 8.35–17.72% with the highest protein contents in the accession line 22,365 (17.72 ± 0.38%). The genotype-725 showed the highest carbohydrate, and dry matter (53.35 ± 0.01 and 93.50 ± 0.07% respectively) contents whereas, the germplasm-830 contained the highest fat (7.88 ± 0.12%) contents while the highest moisture contents were there in germplasm-22348 (11.95 ± 0.06%). The crude fiber contents (19.67 ± 0.19%) were found high in germplasm-832. The mentioned contents were also correlated to each other where a negative (−0.431*) correlation was noted for crude protein and carbohydrate while ash content to crude protein has a positive (0.38*) correlation. A positive and a negative correlation were there in Crude fats/crude protein (0.30*) and crude fats/moisture contents (−0.39*) respectively. Principal component analysis showed an Eigenvalue of 0.76 with a total variation of 85.01% when applied to proximate components. Based on cluster analysis to proximate composition all the oat germplasms were divided into 5 sub-clusters, where accession numbers 769 and 817 were found to be the most diverse genotypes. The elemental analysis confirmed the presence of magnesium (2.89–7.62 mg/L), sodium (3.71–8.03 mg/L), manganese (0.93–3.71 mg/L), copper (0.35–3.36 mg/L), iron (2.15–6.82 mg/L), zinc (1.30–3.37 mg/L), chromium (0.37–3.34 mg/L), and potassium (50.70–59.60 mg/L) in the selected germplasms. Principal component analysis for elemental composition showed the total variation of 73.75% with the Eigenvalue of 0.97. Cluster analysis on an elemental basis divided all the oat germplasms into 7 sub-clusters where accession numbers 769 and 22,350 were found to be the most diverse germplasm. Phytochemical analysis performed through HPLC resulted in the identification of nine possible compounds (malic acid, epigallocatechin gallate, quercetin, morin, ellagic acid, catechin hydrate, rutin, pyrogallol, and mandelic acid) in various germplasm of oat. A concentration-dependent antioxidant response was recorded when extracts were tested as an inhibitor of DPPH and ABTS free radicals. The results revealed that oat grains are a good source of nutrients, minerals, and phytochemicals that can be used as nutraceuticals and as food. The genetic differences revealed that this plant can be grown under varied environmental conditions.

## Graphical abstract

**Figure:**
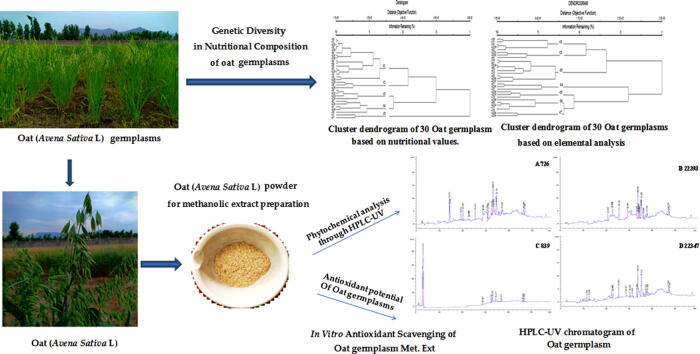

## 1 Introduction

Cereals are cultivated for their nutrient-rich edible grains and are used as food everywhere around the globe. Since the beginning of civilization, certain cereals, such as oat and barley have been used by humans as a staple food, directly or indirectly in terms of livestock feed (Barcchiya et al., 2017). Oats (*Avena sativa* L) are grown all over the world. After wheat, corn, rice, barley, and sorghum, oats are the only grain that is unique among all grain types with rank as sixth in world grain production (Ahmad et al., 2014).

It has been proved experimentally that the phytochemical, elemental, and primary metabolite compositions among different cultivars of the same species are different. To fulfill the human nutrition and health requirements one should select better varieties. Genetic diversity among different cultivars is determined in several ways (Ibrahim et al., 2020). However, in selecting better germplasms with high nutritional values, the determination of diversity for proximate and elemental compositions is a better option. Oats have long been used as a nutritious food. It is a tasty and nutritive food that provides carbohydrates in the form of starch, well-balanced proteins, high lipid levels (Ibrahim et al., 2020). In addition, oatmeal contains small amounts of B vitamins, specifically thiamine, folate, biotin, and pantothenic acid (Gu et al., 2021). Its protein can maintain a good balance of essential amino acids in the body when eaten as food. Many basic amino acids are found in oats, including methionine, cysteine, threonine, isoleucine, tryptophan, valine, leucine, histidine, methionine, phenylalanine, and tyrosine (Biel et al., 2020). Oat crops are commonly used as a companion crop for forage legumes under seedlings. Grains like wheat, maize, barley, and rice provide less protein, fatty acids, iron, magnesium, phosphorus, and zinc in comparison to oat (Gu et al., 2021). In addition, oat is a rich source of fibers (especially β-glucan) and minerals (manganese, phosphorus, copper, iron, selenium, magnesium, and zinc). The bioactive compound present in oats such as phenolics, carotenoids, and sterols is essential for the maintenance of good human health (Ibrahim et al., 2020). It has been reported that oat products in gluten-free and normal diets have a significant role in controlling coeliac disease in humans (Smulders et al., 2018).

The popularity and consumption of oats have increased significantly, however, its output has steadily declined for a few years, especially in Europe (Stewart and McDougall, 2014). Today the food industry is investing significant efforts in increasing the use of oats as an ingredient in the formulation of new food products. The development of new oat products can add more functional foods to the market and would prevent chronic diseases (Jędrusek‐Golińska et al., 2020). In Pakistan, oat is primarily grown as a forage crop on 3.52 million hectares, yielding 264 tons per year. The Punjab province contributes 2.03 million hectares, with oats accounting for more than 35% of the total production (GOP 2014). Oat can tolerate acidic soils better than other small grain cereals showing that the plant has the potential to be grown in varied environments (Chen et al., 2021).

To revive the importance of this plant there is an urgent need to evaluate proximate and elemental composition differences among different cultivars to select the better variety that would have unique health benefits. Therefore, the present study is aimed to figure out proximate and elemental composition differences among high-yielding (30 accessions) oat germplasm out of 236 breeding lines. The most favorable varieties were also subjected to HPLC analysis whereas the antioxidant potentials determined through DPPH and ABTS assays were also evaluated.

## 2 Materials and methods

### 2.1 Plant material

During 2018–2019, 236 genotypes were evaluated for agro morphological and biochemical characterizations in Botanical Garden University of Malakand, Khyber Pakhtunkhwa, Pakistan. The seeds were obtained from Plant Genetic Resources Institute (PGRI) National Agriculture Research Centre Islamabad, Pakistan. About 30 potential high-yielding genotypes were subjected to proximate and elemental composition analysis to get the most potent variety (Table 1).

**Table 1:** Passport information of 30 oat genotypes used in the current study.

**Accession No.**	**Yield (g)**	**Coll_Donor_ No**	**Origin**
A769	26.6	SD CLAV 8421	PGRI Pakistan
A791	26.8	SD CLAV 9201	PGRI Pakistan
A832	26.8	SD PI 468108	PGRI Pakistan
A22329	26.8	MARIDO	PGRI Pakistan
A721	27.4	SD CLAV 8097	PGRI Pakistan
A817	27.6	SD CLAV 9397	PGRI Pakistan
A22335	27.6	AUSTRALIAN	PGRI Pakistan
A841	28	SD PI 479676	PGRI Pakistan
A22349	28.4	BOB	PGRI Pakistan
A22339	28.6	NO.75524	PGRI Pakistan
A739	28.8	SD CLAV 8248	PGRI Pakistan
A22389	28.8	OZARK	PGRI Pakistan
A22393	29	X-5445–4	PGRI Pakistan
A726	29.2	SD CLAV 8170	PGRI Pakistan
A811	29.2	SD CLAV 9316	PGRI Pakistan
A22348	29.8	HAY	PGRI Pakistan
A22365	30.4	S-141	PGRI Pakistan
A725	30.8	SD CLAV 8163	PGRI Pakistan
A839	30.8	SD PI 479658	PGRI Pakistan
A830	31	SD PI 464651	PGRI Pakistan
A672	31.6	–	PGRI Pakistan
A843	32	SD PI 483041	PGRI Pakistan
A22395	32	AVON	PGRI Pakistan
A723	34	SD CLAV 8100	PGRI Pakistan
A22324	34	TAMO	PGRI Pakistan
A22347	34	NO.708	PGRI Pakistan
A22390	35.6	VICAR	PGRI Pakistan
A737	36.4	SD CLAV 8226	PGRI Pakistan
A728	38.2	SD CLAV 8172	PGRI Pakistan
A22350	38.2	NO.2088	PGRI Pakistan

### 2.2 Chemicals and reagents

Chemicals and reagents used in elemental analysis such as H_2_SO_4_, NaOH, HNO_3_, HClO_4_, and C_2_SO_4_ were purchased from Sigma-Aldrich (USA) while Acetic acid from Dae-Jung Korea. The antioxidant chemicals such as DPPH (2, 2-Diphenyl-1-picrylhydrazyl) and ABTS (2, 2-Azino bis [3-ethylbenzthiazoline]-6-sulfonic acid) were purchased from Sigma-Aldrich (St. Louis, MO, USA). Methanol was obtained from Merck: Germany. All chemicals were of analytical grade except HPLC solvents which were of HPLC grade.

### 2.3 Proximate analysis of Avena sativa L. germplasm

About 100 g oat seed samples were finely grounded and subjected to proximate analysis (Horwitz, 2010). In a pre-weighed petri-dish, 3 g of oat powder was weighed and then dried for 6–12 h at 100 °C to determine moisture contents. The dish containing the sample was weighed after cooling in desiccators and differences between the weight before and after moisture contents were estimated.

To determine ash contents, 1 g flour was taken in a crucible which was then placed in a muffle furnace at 550 °C for 5 h. After cooling the crucible weighed. By subtracting crucible weight ash contents were estimated.

To estimate protein contents, the sample (1 g) was digested with concentrated H_2_SO_4_ in the presence of CuSO_4_ and Na_2_SO_4_ and heated. The ammonia produced was steam distilled into a boric acid solution. The ammonia nitrogen was estimated through titration of the trapped ammonia with 0.1 M HCl (double indicator) in presence of Tashirus indicator until the purple-pink color was observed. The deduced nitrogen value was multiplied with a factor of 6.25 mg to get crude protein weight.

For fat contents estimation, 1 g sample of each was extracted with ether (10 mL). Every time tubes were placed for 12 h at 40 °C in the incubator. The upper layer from solutions was transferred to graduated tubes which were then dried in the oven for 4 h at 40 °C to ensure complete evaporation of ether. The weight differences in the tube contents before and after evaporation were considered as fat contents**.**

About 2 g of powder digested in 50 mL of 1.25% H_2_SO_4_ and boil for 30 min. The filtrates were then digested with 50 mL of 1.25% NaOH and heated for 30 min and filtered. The filtrates were then dried in the oven and finally heated to 550 °C in a furnace. The weight of the residue left after the ignition was used to calculate the fiber content, which was expressed in terms of the weight of the sample before ignition. Carbohydrate (CHO %) contents were then estimated from the relation:

(1) CHO%=[100-moisture%-protein%-fat%-ash%]

### 2.4 Atomic Absorption spectrometry

The fine powder of oat seeds was used for the elemental analysis through Atomic Absorption Spectrophotometer (Shimadzu AA-670) following standard methods (Association of Official Analytical Chemists (AOAC) to investigate the mineral composition of oat germplasm (Horwitz, 2010). About 0.25 g of plant material was put in a 50 mL flask containing 6.5 mL mixed acids solution; Nitric acid (HNO_3_), Sulfuric acid (H_2_SO_4_), and Perchloric acid (HClO_4_) in 5:1:0.5 ratios. The samples were boiled on a hot plate in a fume hood (model VWR VELP scientifical, Germany). After that, a few drops of distilled water were added to finish the digestion, which resulted in white fumes coming out of the flask. After cooling the digested samples were then moved into a 50 mL volumetric flask. The volume of the mixture was made 50 mL with distilled water. The samples were then filtered using filter paper; Whatman No. 42 and were stored till further use. The corresponding standard calibration curves were used to calculate the percentage of selected metals.

### 2.5 Sample preparation for HPLC-UV

HPLC profiling was made for 15 samples using an Agilent 1260 system following a reported method (Zeb, 2015). About 1 g powdered sample of oat germplasm was digested with methanol and water (1:1; 20 mL; v/v). The mixture was heated at 70 °C for 1 h in the water bath and centrifuged for 10 min at 4000 rpm. After that sample (2 mL) was filtered into HPLC vials through Whatman filter paper and labeled with proper codes. Identification of phenolic compounds was made through a comparison of sample retention time with that of available standards.

### 2.6 DPPH free radical scavenging

Antioxidant activity of the oat germplasm extract was determined using the Brand William assay (Brand-Williams et al., 1995). To prepare DPPH (2, 2-Diphenyl-1-picrylhydrazyl) solution about 2 mg DPPH was dissolved in 100 mL of methanol. The stock solutions of oat germplasm extract were prepared to have concentrations range 1 mg/mL in methanol and then diluted to the concentrations of 1000, 500, 250, 125, and 62.5 μg/mL. About 0.1 mL diluted solution of each plant part extract was mixed with 3 mL of DPPH solution in methanol and was incubated at 25 °C for 30 min. Finally, the absorbance was measured at 517 nm. Ascorbic acid was used as a positive control. Each concentration was taken in triplicate and the data obtained were presented as mean ± S.E.M. The percent free radical scavenging activity was calculated using the following equation:

(2) $$Percent\;scavenging\;activity\;\text{=}\frac{Contol\;Abs-sample\;Abs}{control\;Abs}\times \;100$$

### 2.7 ABTS free radical scavenging

Antioxidant potentials of the oat germplasm extracts were also assessed using the free radicals of ABTS (2, 2-azino bis [3-ethylbenzthiazoline]-6-sulfonic acid) following standard procedure (Re et al., 1999). The stock solution (1 mg/mL) of oat germplasm extract samples were prepared in methanol and then diluted to the concentration range of 1000, 500, 250, 125, and 62.5 μg/mL. Solutions of ABTS (7 mM) and potassium persulfate (2.45 mM) were prepared and mixed thoroughly. For free radicals' formation, the solution was kept overnight in the dark. After incubation, the absorbance of ABTS solution was adjusted to 0.7 at 745 nm by the addition of methanol (50%). Then 300 μL of test samples were mixed with 3 mL of ABTS solution and then absorbance was measured using a double beam spectrophotometer for 6 min. For positive control ascorbic acid was used. The results were recorded in triplicate and percent ABTS free radicals scavenging activity was calculated using equation (2).

### 2.8 Statistical analysis

All experiments on each oat tested line (germplasm) were performed in three replicates. MS Excel 2016 was used to estimate the mean, cumulative variability (C.V) percent, standard deviation, and other statistics. The mean data of all the studied parameters were subjected to cluster analysis to visualize the cluster dendrogram using linkage analysis, principal component analysis using PC ORD version 5.0, and Statistica version 7. Pearson correlation coefficients were estimated using SPSS version 22.

For antioxidant studies, One-way analysis of variance (ANOVA) followed by Dunnett's posthoc multiple comparison test was used to determine the values of P. P < 0.05 were considered as significant. All the assays were repeated in triplicate and values have been expressed as Mean ± SEM. Linear regression was used to calculate the IC50 values from % inhibition of DPPH and ABTS of different concentrations of oat tested line (germplasm) samples using Excel 2007. (IC50 = half-maximal inhibitory concentration is a measure of the potency of a substance/plant extract in inhibiting free radicals of DPPH and ABTS).

## 3 Results

### 3.1 Proximate contents

In the current study dry matter ranges from 87.60 to 93.50 with the mean value of 90.76, and C.V = 1.71. Minimum dry matter (87.6 ± 0.05) was found in accession number 830, while maximum (93.50 ± 0.07) in accession number-725 (Table 2). A significant variation was also found in crude protein contents ranged from 8.35% to 17.72% (mean value = 14.54%; C. V = 15). Minimum crude protein (8.34 ± 0.03) was found in accession number 726, while maximum (17.72 ± 0.38) in accession −22365. Ash contents were ranged from 3.88 to 8.89 with the mean value of 5.86 and C. V = 24.25. Minimum ash content (3.88 ± 0.01) was there in accession number 22349, while maximum (8.89 ± 0.02) was in accession number 721. Moisture contents were in the range; 7.81–11.95 with the mean value of 6.31 and C.V 11.9. Minimum moisture content was (7.81 ± 0.12) was recorded for accession number 839 while maximum (11.95 ± 0.06) for accession number 22348. Crude fats contents exhibited a significant variation ranging from 4.99 to 7.88 with a mean value of 6.31 and C.V of 13.13. Accession number 811 has shown a minimum quantity of fats (4.99 ± 0.11), while a maximum (7.88 ± 0.12) was found in accession number 830. The crude fibers variations were in the range from 10.27 to 19.67, with a mean value of 15.47, while C.V equal to 16.3. Accession number 22,389 exhibited minimum crude fiber contents (10.27 ± 0.02), while maximum (19.67 ± 0.19) by accession number 832 (Table 2). A significant variation in carbohydrate contents was also observed ranging from 37.52 to 53.40 with the mean value of 47.92 and C.V value of 7.05 with a maximum amount (53.35 ± 0.01) in accession number-725, and minimum (37.51 ± 0.05) in accession number-721.

**Table 2:** Mean value, standard deviation, and descriptive statistics of dry matter, crude protein, ash content, moisture content, crude fats, crude fiber, and carbohydrate of 30 oat germplasm.

**Oat Germplasm**	**Dry matter**	**Crude Protein**	**Ash Content**	**Moisture Content**	**Crude Fats**	**Crude Fiber**	**Carbohydrate**
A769	90.26 ± 0.07	15.57 ± 0.57	4.53 ± 0.03	9.80 ± 0.06	6.95 ± 0.06	15.39 ± 0.02	47.74 ± 0.02
A791	89.64 ± 0.51	13.03 ± 0.08	4.56 ± 0.02	10.75 ± 0.25	6.80 ± 0.06	18.87 ± 0.02	45.96 ± 0.02
A832	91.54 ± 0.28	11.1 ± 0.11	4.02 ± 0.09	8.48 ± 0.15	6.55 ± 0.02	19.67 ± 0.19	50.11 ± 0.02
A22329	90.86 ± 0.11	12.56 ± 0.02	4.77 ± 0.02	9.16 ± 0.01	6.59 ± 0.35	14.14 ± 0.07	52.76 ± 0.01
A721	91.50 ± 0.16	17.71 ± 0.02	8.89 ± 0.02	8.15 ± 0.01	6.20 ± 0.02	16.02 ± 0.08	37.51 ± 0.05
A817	88.67 ± 0.02	14.32 ± 0.05	7.89 ± 0.02	10.35 ± 0.02	7.67 ± 0.02	16.15 ± 0.14	38.94 ± 0.02
A22335	89.67 ± 0.03	14.28 ± 0.01	5.47 ± 0.01	8.67 ± 0.01	7.5 ± 0.08	18.81 ± 0.16	45.27 ± 0.03
A841	92.29 ± 0.06	16.02 ± 0.07	6.34 ± 0.01	8.11 ± 0.12	7.52 ± 0.06	13.32 ± 0.13	48.67 ± 0.02
A22349	93.45 ± 0.01	15.87 ± 0.11	3.88 ± 0.01	8.66 ± 0.10	6.51 ± 0.12	18.77 ± 0.01	46.29 ± 0.02
A22339	91.29 ± 0.17	14.36 ± 0.02	7.61 ± 0.07	8.98 ± 0.11	6.37 ± 0.07	18.78 ± 0.02	43.88 ± 0.02
A739	90.46 ± 0.02	15.48 ± 0.01	8.61 ± 0,07	9.45 ± 0.01	6.65 ± 0.11	14.34 ± 0.02	45.46 ± 0.01
A22389	89.59 ± 0.05	13.26 ± 0.01	7.69 ± 0.16	10.25 ± 0.08	5.45 ± 0.01	10.27 ± 0.02	53.07 ± 0.02
A22393	88.68 ± 0.01	12.17 ± 0.02	4.35 ± 0.02	11.18 ± 0.19	5.10 ± 0.13	17.3 ± 0.16	49.88 ± 0.02
A726	87.71 ± 0.17	8.34 ± 0.03	5.05 ± 0.62	10.67 ± 0.02	5.48 ± 0.05	17.67 ± 0.02	52.78 ± 0.01
A811	93.46 ± 0.02	10.56 ± 0.02	4.67 ± 0.02	11.56 ± 0.02	4.99 ± 0.11	18.54 ± 0.01	49.68 ± 0.01
A22348	92.44 ± 0.31	11.27 ± 0.02	6.37 ± 0.01	11.95 ± 0.06	5.61 ± 0.07	16.67 ± 0.02	48.12 0.01
A22365	91.4 9 ± 0.05	17.72 ± 0.38	8.54 ± 0.02	10.67 ± 0.01	6.37 ± 0.06	12.24 ± 0.02	44.46 ± 0.01
A725	93.50 ± 0.07	14.16 ± 0.02	5.77 ± 0.01	8.77 ± 0.16	6.82 ± 0.06	11.12 ± 0.02	53.35 ± 0.01
A839	92.49 ± 0.11	16.78 ± 0.02	5.99 ± 0.11	7.81 ± 0.12	7.14 ± 0.17	12.23 ± 0.02	50.06 ± 0.02
A830	87.6 ± 0.05	15.85 ± 0.12	5.35 ± 0.01	7.87 ± 0.17	7.88 ± 0.12	14.25 ± 0.02	48.8 ± 0.01
A672	90.41 ± 0.11	15.47 ± 0.02	6.47 ± 0.01	8.91 ± 0.13	5.56 ± 0.19	13.25 ± 0.01	50.3 5 ± 0.01
A843	90.91 ± 0.06	16.72 ± 0.11	5.38 ± 0.01	10.65 ± 0.10	6.59 ± 0.05	13.59 ± 1.15	47.06 ± 0.01
A22395	91.77 ± 0.48	15.50 ± 0.08	4.31 ± 0.08	10.73 ± 0.05	6.10 ± 0.11	15.67 ± 0.02	47.67 ± 0.01
A723	89.52 ± 0.06	16.02 ± 0.07	5.08 ± 0.16	9.7 ± 0.05	6.12 ± 0.01	13.87 ± 0.02	49.18 ± 0.01
A22324	89.70 ± 0.06	16 ± 0.12	6.49 ± 0.06	10.02 ± 0.07	5 ± 0.12	12.13 ± 0.02	50.36 ± 0.02
A22347	89.87 ± 0.18	16.18 ± 0.02	6.43 ± 0.05	8.03 ± 0.08	5.12 ± 0.01	16.08 ± 0.09	48.15 ± 0.01
A22390	90.51 ± 0.06	14.56 ± 0.01	5.75 ± 0.06	8.7 ± 0.06	5.13 ± 0.02	17.27 ± 0.01	48.57 ± 0.01
A737	90.56 ± 0.11	15.67 ± 0.01	6 ± 0.12	9.63 ± 0.12	6.12 ± 0.01	15.56 ± 0.01	46.96 ± 0.04
A728	91.04 ± 0.51	14.49 ± 0.06	5.27 ± 1.05	9.80 ± 0.17	6.38 ± 0.01	16.23 ± 0.01	47.81 ± 0.01
A22350	91.74 ± 0.27	15 ± 1.63	4.33 ± 0.02	9.68 ± 0.08	7.03 ± 0.08	15.92 ± 0.07	48.05 ± 0.02
Descriptive statistics of proximate analysis of 30 oat germplasm							
Range	5.90	9.37	5.01	4.14	2.88	9.40	15.90
Minimum	87.60	8.35	3.88	7.81	5.00	10.27	37.50
Maximum	93.50	17.72	8.89	11.95	7.88	19.67	53.40
Mean	90.76	14.54	5.86	9.58	6.31	15.47	47.92
Standard error	0.28	0.40	0.26	0.21	0.15	0.46	0.66
Standard Deviation	1.56	2.18	1.42	1.14	0.83	2.52	3.60
Variance	2.42	4.76	2.02	1.30	0.69	6.37	12.95
C.V	1.71	15	24.25	11.9	13.13	16.3	7.05

### 3.2 Correlation analysis

The genetic variation among the 30 oat germplasms based on 7 proximate parameters was evaluated (Table 3). Crude protein contents showed a negative correlation with carbohydrate (−0.43*) contents. Ash content was found strongly negative when correlated with carbohydrate contents (−0.48**), and significantly positive (0.38*) with crude protein. For moisture content to crude protein a negative correlation (−0.45*) was recorded. Crude fats were correlated positively with crude protein (0.30*) and negatively with moisture contents (−0.39*). Similarly, the crude fibers were negatively correlated with crude protein (−0.44*) and ash content (−0.49**) respectively.

**Table 3:** Correlation analysis of 30 oat germplasm based on proximate analysis.

**Parameters**	**Carbohydrate**	**Dry Matter**	**Crude Protein**	**Ash Content**	**Moisture Content**	**Crude Fats**
Dry Matter	0.01					
Crude Protein	−0.43*	0.13				
Ash Content	−0.48^**^	−0.07	0.38*			
Moisture Content	0.05	−0.07	−0.45*	−0.11		
Crude Fats	−0.32	0.01	0.30*	−0.06	−0.39*	
Crude Fibers	−0.23	−0.09	−0.44*	−0.49^**^	0.10	−0.07

* and ** means P values (*P < 0.05 and **P < 0.01).

### 3.3 Principal component analysis (PCA)

The principal component analysis (PCA) helps select the best cultivars among a studied population. Table S1 (Supplementary file) shows the PCA (PC1, PC2, PC3, and PC4) of the 7 studied parameters. In the 1st PC, the total variation was 33.78%, and they were associated positively with protein (0.53), ash content (0.45), and fats (0.37) contents, while carbohydrate (−0.46) and fiber (−0.18) correlated negatively.

**Fig. 1:**
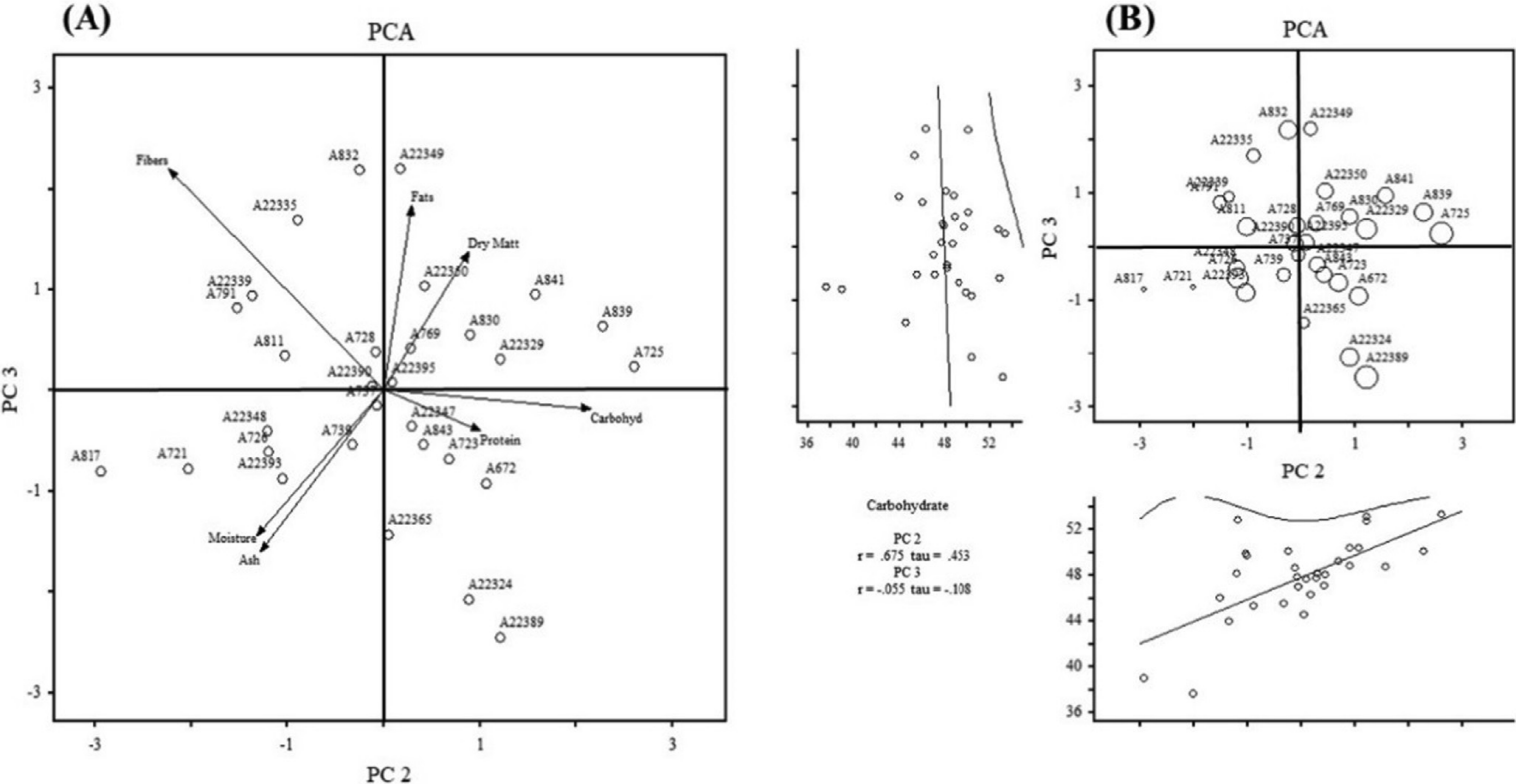
Scatter plot diagram of 30 oat germplasm of PC2 and PC3 of different parameters.

The total variation for the 2nd PC was 54.75% and related positively with carbohydrate (0.56), dry matter (0.23), a protein (0.26) contents while the association of moisture (−0.34), ash (−0.33) and fibers content (−0.58) were negative. In PC3 the total variation was 71.02% and the association of dry matter (0.35), fats (0.47), and fibers (0.57) were recorded positive whereas crude protein (−0.11), ash content (−0.42), and moisture content (−0.38) were found negative. Furthermore, in PC4 the total variation was 85.01%, Eigenvalue was 0.76, and positive scores by weight were exhibited by carbohydrate (0.15) and fats (0.34), while dry matter (−0.88), crude protein (−0.12), ash (−0.11), and moisture contents (−0.26) were found negative. Biplot analysis was used to determine the associations between the traits. As a result, the plot defined by the two PCS: 2 and 3 into 54.75 and 71.02% (Fig. 1) and were placed against each other to find the level of genetic diversity and geographical association among the genotypes. The separation based on PC2 and PC3 revealed that the genotypes were scattered in all the quarters, which showed the highest level of genetic diversity in the studied genotypes, where the accession −817 and 721 have been placed at the left side of the quadrate while 725 and 839 at the right side of the quadrate. The genotypes-672, 723, and 843 have been presented with protein while accession 22,350 with dry matter whereas genotypes-22349 have been marked with fats.

### 3.4 Cluster plotting

**Fig. 2:**
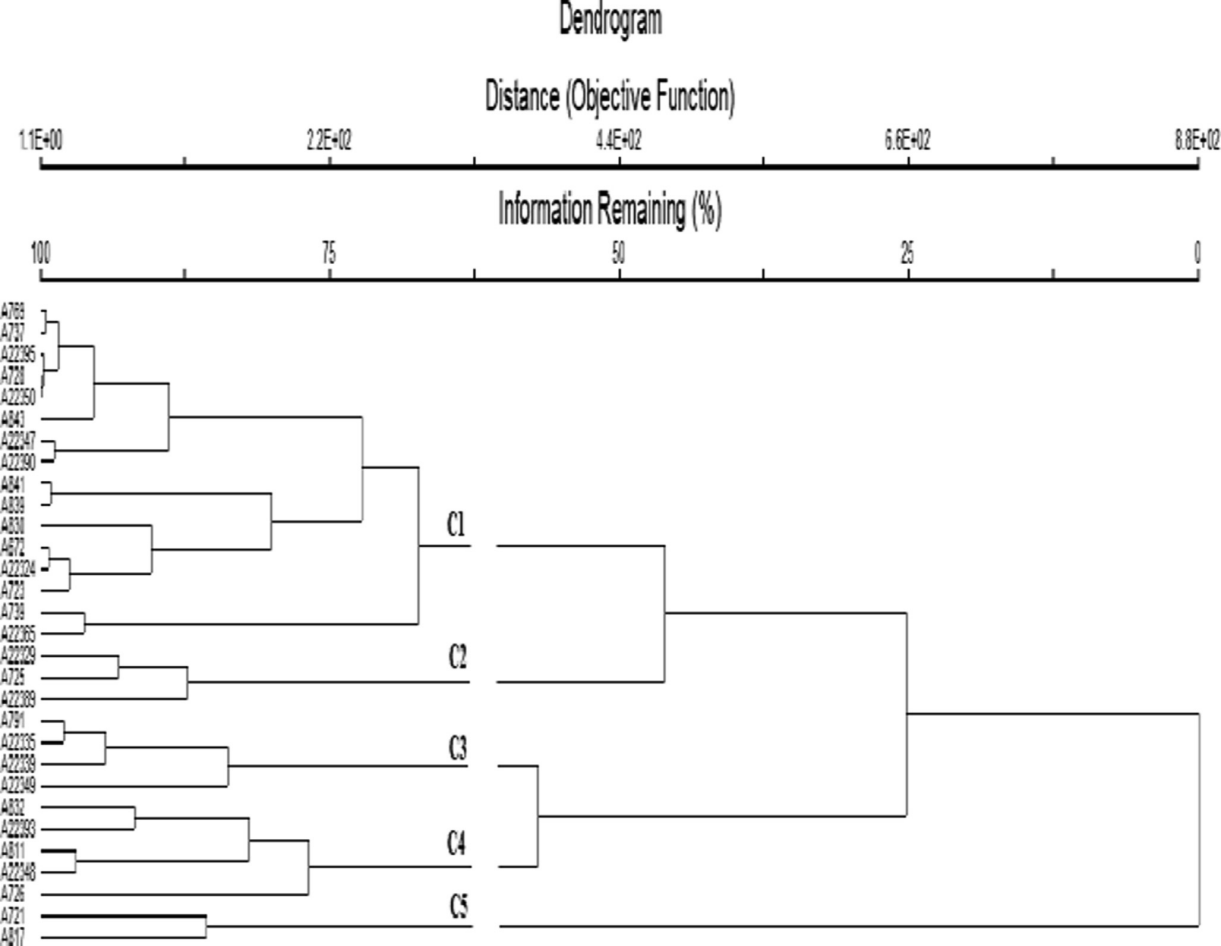
Cluster dendrogram of 30 oat germplasm based on nutritional values.

Wards method has been followed while performing the cluster analysis. All the genotypes have been divided into three main Lineages (lineage 1, lineage 2, and lineage 3) at a distance of 25%, which is further subdivided into 5 sub-clusters at a distance of 62.5% where genotypes 769 and 817 were found the most diverse and were placed at the extreme periphery of the dendrogram. In each group, there was less variation among the genotypes, but they were different from members of the other groups (Fig. 2). The 1st cluster consists of 16 genotypes (769, 737, 22395, 728, 22350, 843, 22347, 22390, 841, 839, 830, 672, 22324, 723, 739 and 22365), while the 2nd sub-cluster consists of three accessions (22329, 725, and 22389). The 3rd cluster comprised of four accessions (791, 22335, 22339, and 22349), whereas the 4th subcluster comprised of 4 genotypes (832, 22393, 811, and 22348). Similarly, the 5th sub-cluster was comprised of 2 accessions: 721 and 817.

The mean performance of all the proximate parameters with various clusters shows that cluster 2 has the highest carbohydrate value of 53.10 ± 0.30 followed by cluster 4 with carbohydrate contents of 49.45 ± 0.91), while cluster 1 (48.11 ± 1.61), and cluster 3 (45.37 ± 1.06) were ranged medium, whereas the lowest carbohydrates contents were found in cluster 5 (38.20 ± 0.98). Cluster 4 has shown the highest dry matter value (91.53 ± 2.05), while lowest protein content (11.29 ± 0.66); cluster 5 has the highest protein (16.01 ± 2.39) and ash contents (8.39 ± 0.70), whereas dry matter was found lowest (90.09 ± 2.00) in this cluster. The fiber contents were recorded highest in cluster 3 (18.81 ± 0.04), followed by cluster 4 (18.04 ± 1.33), while the lowest fibers value (11.85 ± 2.03) was found in cluster 2, followed by cluster 1 (14.71 ± 1.88) (Table S2).

### 3.5 Distribution of minerals elemental composition

The distribution of minerals and elements in 30 oat germplasm are presented in Table 4. Significant variations were observed for the selected elements among the germplasms. Mg ranged from 2.89 to 7.63 mg/L with a mean value of 5.47 and C.V of 23.25%. Maximum Mg (7.62 ± 0.15) was found in accession 830, while minimum (2.88 ± 0.10) in accession 22339. Na ranged from 3.71 to 8.03 mg/L with the mean value of 6.07 and C.V of 20.61%. The maximum quantity of Na (8.03 ± 0.56) was found in accession-22350, while minimum (3.711 ± 0.36) in accession-22365. Mn exhibited values from 0.93 to 3.71 mg/L with a mean value of 1.96 and C.V of 34.64%. Maximum Mn (3.70 ± 0.17) was found in accession number 22390, while minimum (0.92 ± 0.05) in accession-769. K was in the range of 50.70 to 59.60 mg/L, with the mean value of 54.26 and C.V% as 3.69. Accession line 725 displayed a maximum value of K (59.60 ± 5.35), while minimum by 841 (50.7 ± 0.24). Cr was ranged from 0.37 to 3.34 mg/L, with 50.21% C.V and a mean of 1.61. The highest (3.34 ± 0.29) Cr was found in accession number 830, while the lowest (0.36 ± 0.08) was observed in accession line 22393. Zn was in the range of 1.30 to 3.37 mg/L, with a %C.V of 25.34 and a mean value of 2.21. Accession number 726 exhibited maximum Zn (3.37 ± 0.11), while accession number 22,339 displayed minimum (1.30 ± 0.13) Zn contents. Cu exhibited variation from 0.35 to 3.36 mg/L with mean value and %C.V of 1.89 and 45.11, respectively. Maximum Cu (3.35 ± 0.18) was found in accession-723, while minimum (0.35 ± 0.14) in accession 769. Variations in Fe concentrations were from 2.15 to 6.82 mg/L with the mean value of 4.59 and CV% of 31.72%. Maximum Fe (6.81 ± 0.06) was present in accession number 811 while minimum in accession 22,335 (2.14 ± 0.03) as shown in Table 4.

**Table 4:** Mean value, standard deviation, standard error, and coefficient of variation of magnesium, sodium, manganese, potassium, chromium, zinc, copper, and iron of 30 oat germplasms.

Oat Germplasm	Magnesium	Sodium	Manganese	Potassium	Chromium	Zinc	Copper	Iron
A769	4.88 ± 0.12	7.02 ± 0.17	0.92 ± 0.05	53.64 ± 0.44	0.41 ± 0.13	1.83 ± 0.17	0.35 ± 0.14	2.33 ± 0.13
A791	5.13 ± 0.09	4.65 ± 0.13	1.36 ± 0.36	54.01 ± 0.58	1.66 ± 0.12	1.30 ± 0.13	0.44 ± 0.03	2.53 ± 0.11
A832	4.91 ± 0.23	4.11 ± 0.12	1.50 ± 0.06	53.69 ± 0.60	0.63 ± 0.30	2.25 ± 0.17	2.12 ± 0.02	4.17 ± 0.04
A22329	4.97 ± 0.20	4.33 ± 0.11	1.29 ± 0.06	53.51 ± 0.35	0.55 ± 0.10	1.88 ± 0.09	0.68 ± 0.43	3.25 ± 0.07
A721	5.23 ± 0.11	7.57 ± 0.05	2.74 ± 0.27	54.52 ± 0.25	1.30 ± 0.23	2.30 ± 0.15	0.94 ± 0.07	2.2 ± 0.183
A817	6.33 ± 0.10	4.48 ± 0.18	2.02 ± 0.07	55.65 ± 0.46	0.75 ± 0.36	3.30 ± 0.13	0.84 ± 0.16	3.29 ± 0.13
A22335	4.36 ± 0.06	5.56 ± 0.22	2.45 ± 0.11	51.29 ± 0.06	1.06 ± 014	2.52 ± 0.17	2.14 ± 0.02	2.14 ± 0.03
A841	4.35 ± 0.14	7.59 ± 0.07	3.29 ± 0.141	50.7 ± 0.24	1.72 ± 0.43	2.02 ± 0.08	2.87 ± 0.03	4.36 ± 0.15
A22349	3.34 ± 0.15	5.62 ± 0.16	2.45 ± 0.57	55.48 ± 0.17	2.68 ± 0.33	1.51 ± 0.17	0.55 ± 0.11	4.50 ± 0.18
A22339	2.88 ± 0.10	7.51 ± 0.22	2.30 ± 0.14	54.58 ± 0.25	2.15 ± 0.18	1.30 ± 0.13	1.90 ± 0.06	5.22 ± 0.10
A739	3.29 ± 0.14	6.32 ± 0.06	1.80 ± 0.22	52.72 ± 0.74	2.29 ± 0.23	1.95 ± 0.06	0.95 ± 0.06	6.23 ± 0.11
A22389	4.36 ± 0.06	7.35 ± 0.06	1.44 ± 0.12	58.62 ± 0.12	2.25 ± 0.16	2.30 ± 0.16	2.15 ± 0.02	5.39 ± 0.08
A22393	4.95 ± 0.05	7.47 ± 0.07	1.76 ± 0.21	51.93 ± 0.66	0.36 ± 0.08	3.29 ± 0.14	3.02 ± 0.09	2.16 ± 0.01
A726	4.59 ± 0.06	5.81 ± 0.05	2.156 ± 0.03	54.07 ± 0.45	0.95 ± 0.06	3.37 ± 0.11	1.68 ± 0.51	6.66 ± 0.32
A811	5.29 ± 0.16	5.59 ± 0.12	3.02 ± 0.17	52.62 ± 0.31	1.33 ± 0.11	3.31 ± 0.10	1.77 ± 0.21	6.81 ± 0.06
A22348	5.47 ± 0.07	6.59 ± 0.17	1.78 ± 0.09	52.96 ± 0.88	1.08 ± 0.89	2.29 ± 0.14	2.27 ± 0.24	5.46 ± 0.06
A22365	6.43 ± 0.10	3.71 ± 0.36	2.20 ± 0.10	56.69 ± 0.24	1.38 ± 0.45	2.97 ± 0.11	2.57 ± 0.33	4.41 ± 0.13
A725	7.40 ± 0.06	4.10 ± 0.11	2.22 ± 0.11	59.60 ± 5.35	2.7 ± 0.27	1.68 ± 0.51	2.31 ± 0.31	3.15 ± 0.06
A839	5.70 ± 0.05	5.65 ± 0.22	1.03 ± 0.18	55.51 ± 013	3.04 ± 0.56	1.94 ± 0.05	2.5 ± 0.41	5.02 ± 0.07
A830	7.62 ± 0.15	5.47 ± 0.18	1.03 ± 0.18	52.64 ± 0.16	3.34 ± 0.29	1.77 ± 0.20	2.22 ± 0.08	5.39 ± 0.06
A672	6.62 ± 0.14	6.35 ± 0.06	2.25 ± 0.17	51.52 ± 0.27	1.81 ± 0.43	2.41 ± 0.27	3.30 ± 0.13	5.68 ± 1.19
A843	5.745 ± 0.07	7.03 ± 0.16	2.33 ± 0.22	53.91 ± 0.12	0.96 ± 0.06	1.94 ± 0.04	3.11 ± 0.12	5.18 ± 0.11
A22395	6.81 ± 0.06	5.87 ± 0.10	1.03 ± 0.17	52.49 ± 0.38	1.94 ± 0.05	2.31 ± 0.31	2.32 ± 0.29	4.49 ± 0.16
A723	6.88 ± 0.40	4.75 ± 0.07	2.03 ± 0.08	54.66 ± 0.10	0.96 ± 0.03	2.238 ± 0.18	3.35 ± 0.18	5.70 ± 0.07
A22324	5.51 ± 0.14	5.84 ± 0.45	1.52 ± 0.08	55.66 ± 0.20	1.48 ± 0.17	1.95 ± 0.06	1.55 ± 0.12	4.40 ± 0.11
A22347	7.48 ± 0.24	5.87 ± 1.06	1.73 ± 0.17	53.96 ± 0.17	1.96 ± 0.01	2.20 ± 0.09	2.03 ± 0.09	3.29 ± 0.11
A22390	5.90 ± 0.06	8.02 ± 0.16	3.70 ± 0.17	55.93 ± 0.91	2.59 ± 0.41	2.32 ± 0.28	1.11 ± 0.12	6.51 ± 0.14
A737	5.62 ± 0.18	7.05 ± 0.13	1.81 ± 0.15	55.61 ± 0.19	0.98 ± 0.01	1.97 ± 0.01	2.11 ± 0.09	5.77 ± 0.09
A728	4.43 ± 0.12	6.62 ± 0.13	1.25 ± 0.18	55.85 ± 0.51	1.45 ± 0.29	1.80 ± 0.15	1.93 ± 0.04	5.55 ± 0.19
A22350	7.60 ± 0.42	8.03 ± 0.56	2.23 ± 0.19	53.82 ± 0.46	2.64 ± 0.42	2.01 ± 0.08	1.51 ± 0.17	6.59 ± 0.26
**Descriptive statistics of elemental analysis of 30 oat germplasm**								
Range	4.74	4.32	2.78	8.90	2.97	0.07	3.01	4.67
Minimum	2.89	3.71	0.93	50.70	0.37	1.30	0.35	2.15
Maximum	7.63	8.03	3.71	59.60	3.34	3.37	3.36	6.82
Mean	5.47	6.07	1.96	54.26	1.61	2.21	1.89	4.59
Standard error	0.23	0.22	0.12	0.36	0.14	0.10	0.15	0.26
Standard Deviation	1.27	1.25	0.67	2.00	0.81	0.56	0.85	1.45
CV%	23.25	20.61	34.64	3.69	50.21	25.34	45.11	31.72

### 3.6 PCA for elemental composition

**Fig. 3:**
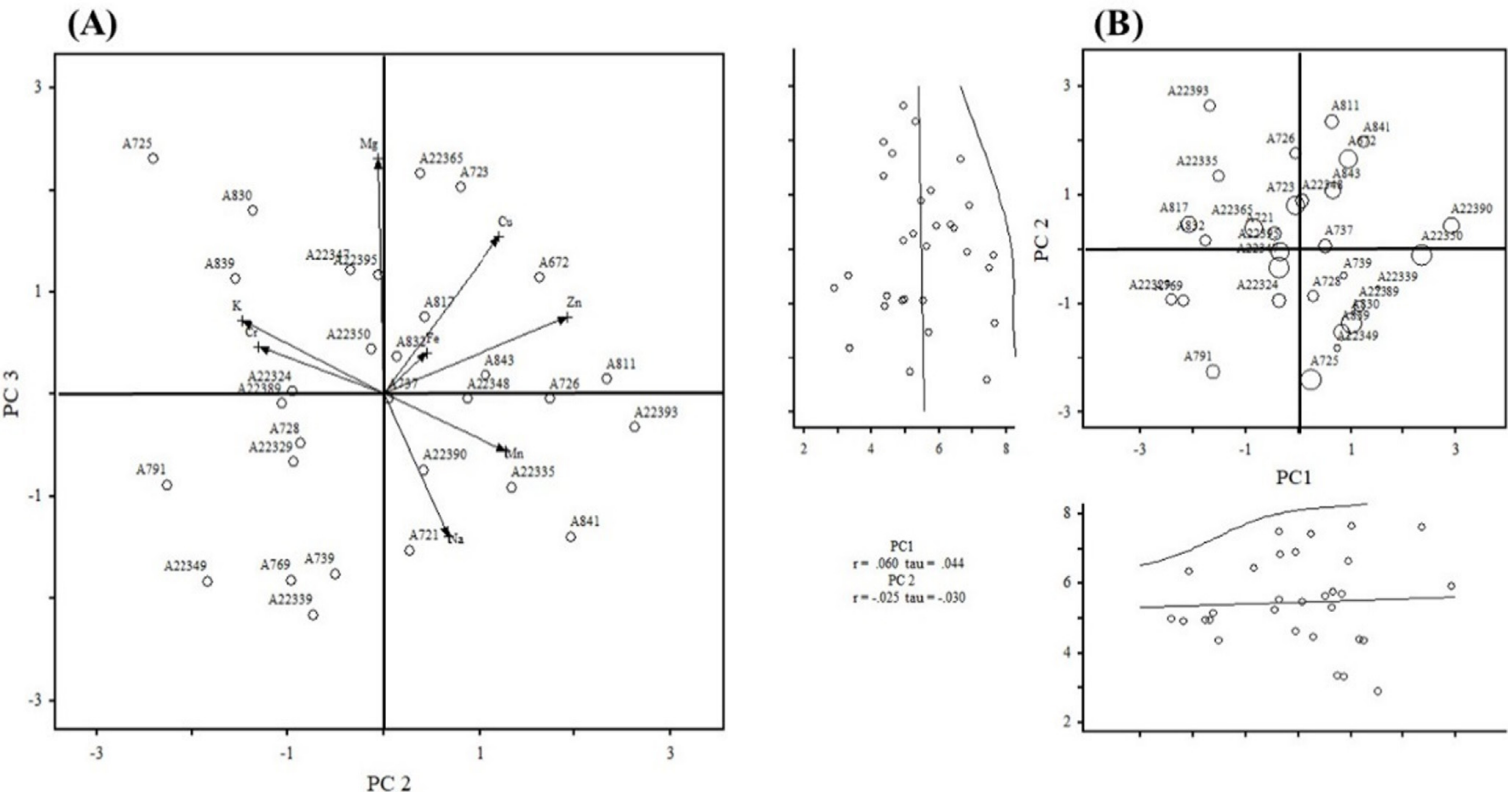
Scatter plot diagram of 30 oat germplasms of elemental analysis of PC2 and PC3.

Principle component (PC1, PC2, PC3, and PC4) analysis for the elements like magnesium, sodium, manganese, potassium, chromium, zinc, copper, and iron distribution in selected germplasms were performed where a total variation of 73.75% and Eigenvalue of 0. 97 (Table S3) were recorded. PC1 accounted for a total variation of 21.42%, the germplasm with a positive effect for sodium (0.42), manganese (0.34), potassium (0.10), chromium (0.54), and iron (0.57) while negative effects by weight for zinc (−0.17). The oat germplasms in PC2 showed positive values for sodium (0.20), manganese (0.37), zinc (0.57), copper (0.36), and iron (0.13), while negative in the case of potassium (−0.43) and chromium (−0.38) with the total variations of 42.70%. The PC3 depicted 1.51 Eigenvalue and 61.62% of the variability. The oat germplasms in PC3 presented positive results for magnesium (0.68), potassium (0.21), chromium (0.13), zinc (0.22), copper (0.45), and iron (0.19), while negative results were observed in the case of sodium (−0.41) and manganese (−0.17) with the total variation 61.62%. The PC4 revealed 73.75% of the variability. The oat germplasms in PC4 exhibited positive effects for sodium (0.24), chromium (0.06), and copper (0.38), while negative in the case of manganese (−0.48), potassium (−0.61), zinc (−0.40), and iron (−0.08) with total percent variations of 73.75. The scatter biplot analysis showed that the two PCS: PC2 (42.70%) and PC3 (61.62%) variation and were placed against each other to find the level of genetic diversity and geographical association among the genotypes. The separation based on PC2 and PC3 revealed that the genotypes were scattered in all the quarters, which showed a high level of genetic diversity in the studied genotypes. That was observed for the genotype-725 in terms of Mg contents. The genotype 22,350 exhibited almost equal in terms of Cr and Fe. The genotypes- 817, 832, and 22,393 were found at the left side of the quadrates while 22,390 was at the right side of the quadrates. The genotypes-841, 739, 22,349, and 22,339 were found highest in Na (Fig. 3; Table S3).

### 3.7 Cluster analysis for elemental composition

**Fig. 4:**
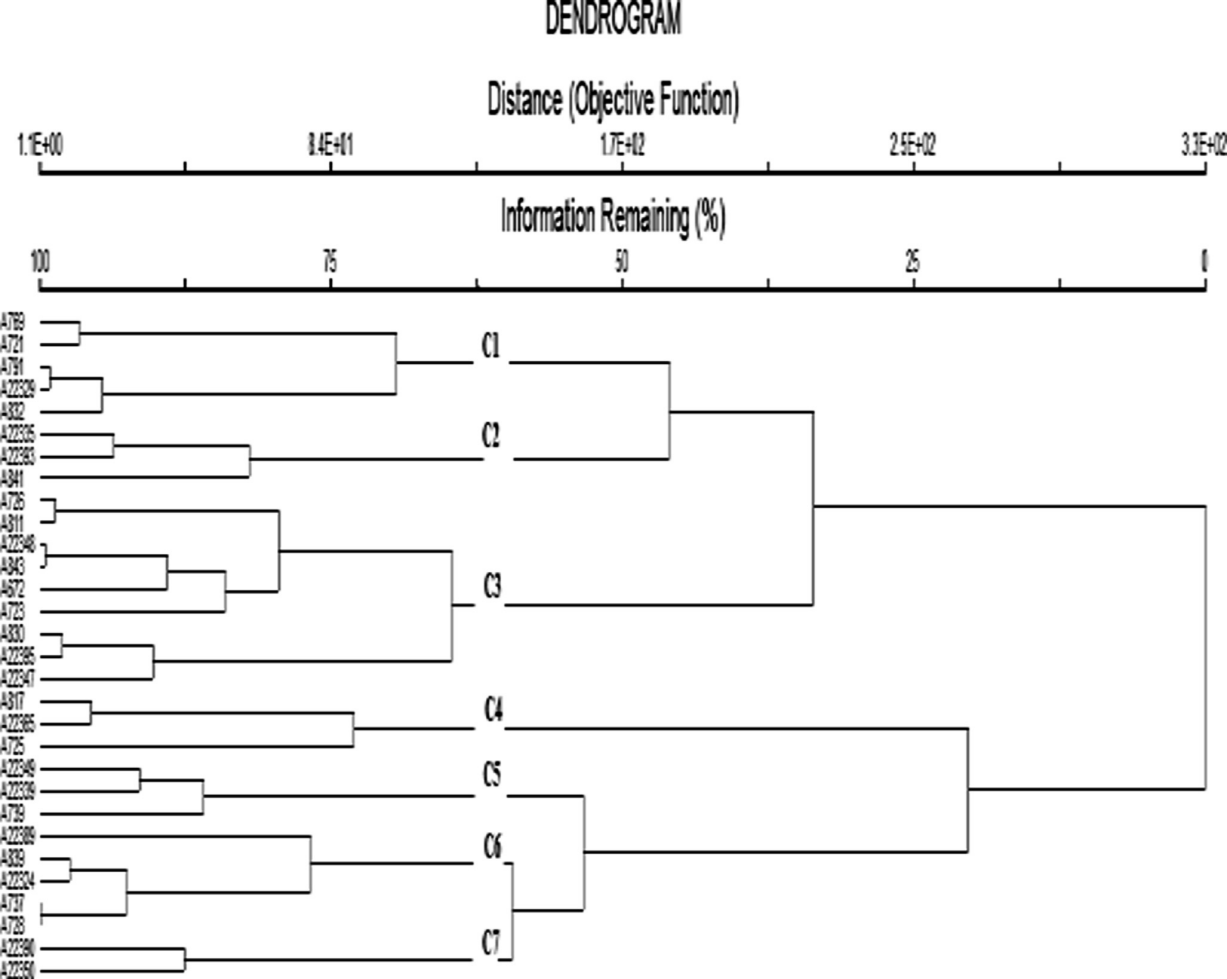
Cluster dendrogram of 30 oat germplasms based on elemental analysis.

Cluster analysis based on elemental analysis was performed following Ward’s method of variance which divided all the oat germplasm into three main Lineages; Lineage 1, Lineage 2, and Lineage 3 which were further divided into 7 sub-clusters. Accession numbers 769 and 22,350 were found to be the most diverse germplasm among the studied germplasms. Each group has shown less variation within the group but exhibited more variation from the other groups (Fig. 4, Table S4). The 1st cluster was comprised of 5 accessions; 769, 721, 791, 22,329, and 832, while the 2nd sub-cluster of three accessions; 22335, 22,393, and 841. The 3rd cluster was composed of 9 accessions (726, 811, 22348, 843, 672, 723, 830, 22,395, and 22,347), whereas 3 (each) accession lines were there in cluster 4 (817, 22,365, and 725) and 5 (22,349, 22,339, and 739). The sub-cluster 6th consisted of 5 accessions-22389, 839, 22,324, 737, and 728, while the 7th sub-cluster was composed of 2 accessions: 22,390 and 22,350. The comparative study showed that cluster 7 accessions have the highest magnesium (6.75 ± 1.19), whereas the lowest (3.17 ± 0.24) was recorded in accession of cluster 5. The highest sodium contents (8.03 ± 0.01) were found in the accessions of cluster 7 and the lowest (4.1 ± 0.38) in cluster 4 accessions. Furthermore, the cluster 7 accessions also exhibited the highest manganese (2.97 ± 1.04), while the lowest (1.41 ± 0.29) was found in cluster 6 lines. The highest potassium (56.25 ± 1.33) was found in cluster 6, whereas a low level (51.31 ± 0.61) was found in cluster 2. Cluster 7 displayed the highest chromium contents (2.62 ± 0.35), while the lowest value was recorded for cluster 1 (0.91 ± 0.53). In addition, the highest value of zinc was found in accession of cluster 4 (2.65 ± 0.85), while the lowest (1.59 ± 0.33) in cluster 5. The highest (6.55 ± 0.05) quantity of iron was found in the accession of cluster 7, while the lowest (2.89 ± 1.27) in cluster 2 (Table S4).

### 3.8 HPLC-UV chromatograms

Typical HPLC-UV chromatograms of *Avena sativa* L. (oat) germplasms are presented in Fig. S1-S15 (Supplementary file). Two compounds (malic acid and pyrogallol) were identified in accession 832, while three phytochemicals (malic acid, pyrogallol, and mandelic acid) were identified in 791 (Fig. S1 & S2). A total of four phenolic compounds; rutin, pyrogallol, mandelic acid, and epigallocatechin gallate were identified in 672 (Fig. S3), while seven (malic acid, epigallocatechin gallate, quercetin, morin, rutin, pyrogallol, and mandelic acid) in line 22,348 (Fig. S4). Accession-726 contains three compounds: catechin hydrate, rutin, and mandelic acid (Fig. S5), while in 22,393, 3 compounds (catechin hydrate, rutin, mandelic acid) were identified (Fig. S6). Accession-839 has two compounds (malic acid and mandelic acid) while in line 22,347; morin, rutin, and mandelic acid (Fig. S7 & S8) were identified. Seven phytochemicals were identified in line 811 (malic acid, epigallocatechin gallate, quercetin, morin, rutin, pyrogallol, and mandelic acid (Fig. S9). Three compounds in line 830 (morin, catechin hydrate, and mandelic acid) were confirmed whereas in line 841 also 3 compounds; catechin hydrate, pyrogallol, and mandelic acid were there (Fig. S10 & S11). Epigallocatechin gallate, morin, ellagic acid, rutin, and pyrogallol were identified from the chromatogram of accession-843 (Fig. S12), while three phytochemicals (catechin hydrate, rutin, and pyrogallol) were identified in accession-22,390 (Fig. S13). Catechin hydrate, pyrogallol, and mandelic acid were identified from chromatogram of accession-22,349 (Fig. S14), while in line 22,335 four phytochemicals compounds like malic acid, morin, catechin hydrate, and pyrogallol (Fig. S15) were identified. The quantification and identification of each phenolic compound with its particular peak position and retention time (Rt) in the chromatogram is presented in Table 5.

**Table 5:** Identification and quantification of phenolic phytochemical compounds in oat germplasm.

**Oat Germplasm**	**No. of Peak**	**Retention time (min)**	**Phenolic compounds Identity**	**HPLC-UV λmax (nm)**	**Peak Area of sample**	**Peak Area of standard**	**Concentration (µg/ml)**	**Identification Reference**
A832	1	2.3	Malic acid	320	971.440	40.32	21.68	Reference Standard
	2	28.0	Pyrogallol	320	32.004	1.014	28.40	Reference Standard
A791	1	2.3	Malic acid	320	636.477	40.32	14.21	Reference Standard
	2	28.0	Pyrogallol	320	99.156	1.014	88.01	Reference Standard
	3	30.2	Mandelic acid	320	132.752	72.0	1.65	Reference Standard
A672	1	12.4	Morin	320	35.706	20.0	1.606	Reference Standard
	2	22.6	Rutin	320	364.509	2241.2	0.146	Reference Standard
	3	28.0	Pyrogallol	320	34.477	1.014	30.601	Reference Standard
	4	30.2	Mandelic acid	320	58.098	72.0	0.726	Reference Standard
A22348	1	8.5	Epigallocatechin gallate	320	56.483	7261.47	0.007	Reference Standard
	2	12.4	Morin	320	70.389	20.0	3.167	Reference Standard
	3	16.5	Ellagic acid	320	217.487	319.24	0.613	Reference Standard
	4	20.5	Catechin hydrate	320	19.210	78.0	0.222	Reference Standard
	5	22.6	Rutin	320	1422.771	2241.2	0.571	Reference Standard
	6	28	Pyrogallol	320	237.006	1.014	210.360	Reference Standard
	7	30.2	Mandelic acid	320	138.769	72.0	1.734	Reference Standard
A726	1	20.5	Catechin hydrate	320	44.733	78.0	0.516	Reference Standard
	2	22.6	Rutin	320	20.326	2241.2	0.900	Reference Standard
	3	30.2	Mandelic acid	320	55.450	72.0	0.639	Reference Standard
A22393	1	20.5	Catechin hydrate	320	27.131	78.0	0.313	Reference Standard
	2	22.7	Rutin	320	20.326	2241.2	0.008	Reference Standard
	3	30.2	Mandelic acid	320	39.318	72.0	0.491	Reference Standard
A839	1	2.3	Malic acid	320	1640.939	40.323	36.625	Reference Standard
	2	30.2	Mandelic acid	320	31.719	72.0	0.396	Reference Standard
A22347	1	12.4	Morin	320	18.108	20.0	0.814	Reference Standard
	2	22.6	Rutin	320	31.765	2241.2	0.013	Reference Standard
	3	30.2	Mandelic acid	320	59.845	72.0	0.748	Reference Standard
A811	1	2.3	Malic acid	320	25.140	40.32	0.561	Reference Standard
	2	8.0	Epigallocatechin gallate	320	59.186	7261.47	0.007	Reference Standard
	3	10	Quercetin	320	336.800	7089.28	0.043	Reference Standard
	4	12.4	Morin	320	97.392	20.0	4.382	Reference Standard
	5	22.7	Rutin	320	130.549	2241.2	0.052	Reference Standard
	6	28.0	Pyrogallol	320	61.522	1.014	54.605	Reference Standard
	7	30.2	Mandelic acid	320	23.036	72.0	0.287	Reference Standard
A830	1	12.4	Morin	320	51.158	20.0	2.302	Reference Standard
	2	20.5	Catechin hydrate	320	53.086	78.0	0.613	Reference Standard
	3	30.2	Mandelic acid	320	52.425	72.0	0.655	Reference Standard
A841	1	20.5	Catechin hydrate	320	31.531	78.0	0.364	Reference Standard
	2	28.0	Pyrogallol	320	71.173	1.014	63.171	Reference Standard
	3	30.2	Mandelic acid	320	99.571	72.0	1.244	Reference Standard
A843	1	8.4	Epigallocatechin gallate	320	97.547	7261.47	0.012	Reference Standard
	2	12.4	Morin	320	113.804	20.0	5.121	Reference Standard
	3	16.4	Ellagic acid	320	341.521	319.24	0.963	Reference Standard
	4	22.6	Rutin	320	2185.414	2241.2	0.877	Reference Standard
	5	28.0	Pyrogallol	320	368.042	1.014	326.663	Reference Standard
A22390	1	20.5	Catechin hydrate	320	67.223	78.0	0.775	Reference Standard
	2	22.6	Rutin	320	25.955	2241.2	0.010	Reference Standard
	3	28	Pyrogallol	320	57.917	1.014	51.405	Reference Standard
A22351	1	20.5	Catechin hydrate	320	21.615	78.0	0.249	Reference Standard
	2	28.0	Pyrogallol	320	30.947	1.014	27.467	Reference Standard
	3	30.2	Mandelic acid	320	22.912	72.0	0.286	Reference Standard
A22335	1	2.3	Malic acid	320	33.067	40.32	0.738	Reference Standard
	2	12.4	Morin	320	18.708	20.0	0.841	Reference Standard
	3	20.5	Catechin hydrate	320	70.602	78.0	0.814	Reference Standard
	4	28.0	Pyrogallol	320	62.396	1.014	55.381	Reference Standard

### 3.9 Antioxidant activity

#### 3.9.1 DPPH free radical inhibition

DPPH scavenging activity of s oat germplasms Met. Ext is presented in Table 6. The DPPH scavenging potential of methanolic extracts of accession line 22,347 was highest with an IC_50_ value of 20 µg/mL, followed by lines 22,348 and 843 with IC_50_ of 30 and 32 µg/mL respectively as shown in Table 6. Ascorbic acid was used as the standard for which the IC_50_ value was 15 µg/mL.

**Table 6:** Percent DPPH and ABTS free radical scavenging activity of Me. Ext of oat germplasm at various concentrations.

**S. No**	**Oat Germplasm**	**Concentration (µg/ml)**	**%DPPH scavenging**	**IC_50_ (µg/ml)**	**%ABTS scavenging**	**IC_50_ (µg/ml)**
			Mean ± SEM		Mean ± SEM	
1	Met. Ext-832	1000	55.40 ± 1.1 ***	500	50.33 ± 0.6***	510
		500	50.14 ± 0.7 ***		48.12 ± 0.7***	
		250	45.32 ± 0.5 ***		45.21 ± 1.1***	
		125	32.11 ± 0.6 ***		35.84 ± 0.9***	
		62.5	30.45 ± 1.0***		31.25 ± 0.2***	
		31.05	28.74 ± 0.7 ***		29.55 ± 0.9***	
2	Met. Ext-791	1000	62.30 ± 0.6***	240	57.12 ± 0.7***	360
		500	55.14 ± 0.5 ***		51.44 ± 0.1***	
		250	50.12 ± 0.4***		48.01 ± 0.6***	
		125	43.23 ± 0.5***		42.36 ± 0.4***	
		62.5	38.45 ± 0.3***		34.55 ± 0.6***	
		31.05	33.23 ± 0.7***		29.34 ± 1.0***	
3	Met. Ext- 672	1000	78.19 ± 1.7 **	40	81.21 ± 0.5*	33
		500	73.23 ± 0.4**		74.12 ± 0.7**	
		250	69.85 ± 1.5**		68.45 ± 0.3**	
		125	62.12 ± 0.1**		62.31 ± 0.7**	
		62.5	55.14 ± 0.2**		54.12 ± 1.2**	
		31.05	47.23 ± 0.9**		49.85 ± 1.1 **	
4	Met.Ext-22348	1000	89.12 ± 0.2 *	30	84.55 ± 0.7*	32
		500	82.12 ± 0.4**		80.45 ± 0.6*	
		250	77.56 ± 1.2**		75.12 ± 0.4**	
		125	73.22 ± 0.7*		69.12 ± 1.0*	
		62.5	66.52 ± 0.7**		63.17 ± 0.9**	
		31.05	50.23 ± 0.9 **		49.33 ± 0.8**	
5	Met. Ext- 726	1000	73.89 ± 1.1***	45	70.23 ± 0.29***	65
		500	70.12 ± 1.6***		65.23 ± 0.1***	
		250	65.13 ± 0.8***		62.13 ± 0.6***	
		125	60.15 ± 0.4 ***		56.25 ± 0.7***	
		62.5	55.42 ± 0.9***		49.20 ± 1.3***	
		31.05	45.12 ± 1.1***		44.71 ± 1.1***	
6	Met.Ext-22393	1000	73.36 ± 1.2***	65	70.12 ± 0.5***	90
		500	68.23 ± 0.7 ***		63.24 ± 0.5***	
		250	60.12 ± 0.6***		59.82 ± 0.6***	
		125	55.41 ± 0.5***		54.23 ± 0.4***	
		62.5	48.21 ± 0.7***		45.26 ± 1.2***	
		31.05	42.13 ± 0.4***		40.12 ± 0.3***	
7	Met. Ext-839	1000	55.31 ± 1.2***	500	63.12 ± 0.5***	200
		500	50.12 ± 0.7***		55.23 ± 0.1***	
		250	45.41 ± 0.6***		51.23 ± 0.6***	
		125	40.12 ± 0.5***		47.23 ± 0.9***	
		62.5	37.22 ± 0.7***		39.63 ± 1.0***	
		31.05	33.22 ± 0.9***		35.24 ± 0.5***	
8	Met.Ext-22347	1000	89.36 ± 1.1*	20	87.55 ± 0.59*	25
		500	85.32 ± 0.7 *		82.14. ± 0.51*	
		250	80.21 ± 0.6 *		79.23 ± 0.6*	
		125	77.42 ± 0.5 *		74.23 ± 0.7*	
		62.5	69.23 ± 0.7 *		65.33 ± 1.0*	
		31.05	58.23 ± 0.7 *		56.52 ± 1.0*	
9	Met. Ext- 811	1000	70.36 ± 1.1***	55	68.55 ± 0.5***	100
		500	66.33 ± 0.6 ***		64.12 ± 0.5***	
		250	60.23 ± 0.6 ***		55.25 ± 0.6***	
		125	57.33 ± 0.5 ***		51.33 ± 0.7***	
		62.5	51.25 ± 0.7 ***		47.63 ± 1.9***	
		31.05	44.25 ± 0.8 ***		41.15 ± 0.5***	
10	Met. Ext- 830	1000	56.69 ± 0.98 ***	500	55.45 ± 0.55***	500
		500	50.12 ± 1.21***		50.12 ± 0.5***	
		250	44.25 ± 0.7***		48.71 ± 0.2***	
		125	37.12 ± 0.6***		41.23 ± 0.6***	
		62.5	32.23 ± 0.5***		35.63 ± 0.7***	
		31.05	29.88 ± 0.7***		27.12 ± 1.3***	
11	Met. Ext- 841	1000	57.15 ± 0.4 ***	360	55.14 ± 1.0***	440
		500	52.12 ± 0.1***		51.12 ± 0.4***	
		250	47.52 ± 1.5***		45.33 ± 0.6***	
		125	43.23 ± 0.4***		39.88 ± 0.5***	
		62.5	38.55 ± 0.6***		37.41 ± 0.9***	
		31.05	33.63 ± 0.7***		33.45 ± 1.2***	
12	Met. Ext- 843	1000	73.45 ± 0.5 ***	32	70.12 ± 0.9***	35
		500	69.88 ± 0.7 ***		66.63 ± 1.9***	
		250	64.24 ± 0.3***		60.33 ± 0.5***	
		125	59.85 ± 1.2***		57.23 ± 0.5***	
		62.5	54.14 ± 0.7***		54.12 ± 0.1***	
		31.05	50.22 ± 0.5 **		49.85 ± 1.9**	
13	Met.Ext-22390	1000	66.14 ± 0.1 ***	90	60.55 ± 0.6***	160
		500	62.33 ± 0.5 ***		58.52 ± 0.8***	
		250	57.23 ± 0.7 ***		53.63 ± 1.2***	
		125	52.13 ± 0.9***		48.25 ± 0.5***	
		62.5	48.33 ± 1.3***		44.45 ± 0.4***	
		31.05	44.74 ± 0.4***		39.66 ± 1.2***	
14	Met.Ext-22351	1000	67.47 ± 0.76 ***	85	60.35 ± 0.1***	270
		500	63.25 ± 0.61 ***		58.55 ± 0.8***	
		250	55.88 ± 0.55 ***		54.71 ± 0.7***	
		125	51.15 ± 0.78 ***		46.32 ± 1.0***	
		62.5	49.88 ± 0.98 ***		39.58 ± 0.4***	
		31.05	41.25 ± 0.78 ***		37.41 ± 1.3***	
15	Met.Ext-22335	1000	70.36 ± 1.3***	60	71.12 ± 1.4***	65
		500	68.55 ± 0.9 ***		68.55 ± 1.1***	
		250	62.33 ± 0.5 ***		63.15 ± 1.5***	
		125	56.12 ± 0.5 ***		59.55 ± 0.6***	
		62.5	50.41 ± 0.6 ***		50.66 ± 1.5***	
		31.05	47.23 ± 0.2 ***		44.78 ± 1.0***	
16	Ascorbic acid	1000	92.33 ± 0.2	15	90.25 ± 0.4	18
		500	89.55 ± 0.3		88.55 ± 0.5	
		250	84.71 ± 1.0		83.55 ± 0.9	
		125	78.33 ± 0.9		79.23 ± 0.8	
		62.5	70.12 ± 0.7		73.55 ± 0.1	
		31.05	65.33 ± 0.4		64.52 ± 0.6	

Met. Ext, methanolic extract. Note: The data is represented as mean ± SEM, (n = 3), *P < 0.05, **P < 0.01, ***P < 0.001; comparison of Me. Ext of oat germplasm vs positive control Ascorbic acid.

#### 3.9.2 ABTS free radical screening

ABTS scavenging activity of oat germplasms Met. Ext is presented in Table 6. Accession line 22,347 was found more potent with an IC_50_ value of 25 µg/mL, followed by lines 22,348 and 843 with IC_50_ of 32 and 35 µg/mL, respectively. For ascorbic acid, the IC_50_ value was 18 µg/mL.

## 4 Discussion

Interest in oats has recently increased due to their nutritional value and health benefits. Macronutrients (fats, proteins, carbohydrates) provide energy and substances necessary for body growth, while micronutrients (vitamins and minerals) aid in the successful operation of metabolic pathways (Hans and Jana 2018). Secondary metabolites are substances prepared by plants in an uneven situation to protect themselves from enemies or diseases. The knowledge of the nutritional and phytochemical constituents of a plant is particularly important for humans as they are directly or indirectly dependent on plants. In this connection, 30 oat germplasms were evaluated for nutritional (proximate), elemental, and phytochemical compositions.

Carbohydrates account for over 70% of the dry weight of cereals and are the most essential ingredient. A significant variation was found in carbohydrate contents with a minimum value in accession number-721, while the maximum was recorded for accession-725. At the same time, dry matter is ranged from 87.60% to 93.50% showing a connection with carbohydrate contents i.e. greater the carbohydrate contents greater will be the amount of dry matter. The finding shows that all varieties do not have the same amount of carbohydrates, being a primary metabolite. Thus accession-725 will be an excellent choice for breeders as oat flour could be a useful source of carbohydrates. Youssef et al., (2016) reported that the carbohydrates content of different oat genotypes can vary from 69.43 to 75.62%. According to Punia et al., (2020) starch is the most important digestible carbohydrate of plants, which is thus an important source of energy for humans and animals. The range of carbohydrate content varies in different oat varieties and as an example, few reported values are given as; 45.20–53.61% (Kose, Mut, & Akay, 2021), 45.7–46.3% (Brunava et al., 2014), and 42.7–49.6% (Mut et al., 2017).

Oat is the potential source of proteins as it possesses high protein contents with a unique composition like; a high proportion of salt soluble globulins, which are endosperm storage proteins, whereas storage proteins in other cereals are insoluble in salt solutions (Mut et al., 2017). We observed a significant variation in protein content among the studied germplasms (8.35–17.72%), where maximum contents were recorded in accession-22365. Protein is an important indicator to measure the quality of oat grains. The protein content depends on environmental factors as well as genotypic factors. Prates and Yu (2017) recorded protein content in several oat germplasms which were from 148.7 to 216.6 mg/g. In another study, the contents recorded were from 30 to 280 mg/g (Bhardwaj et al., 2019), which is the highest value reported so far.

The range of fat contents recorded was from 4.99% to 7.88% with a minimum value in accession number 811 and maximum in accession-830. In the study of Sterna, Zute, & Brunava, (2016) the fat contents were ranged from 4.2 to 11.8%. Kose et al., (2021) have reported the fat, linolenic, oleic, palmitic, and stearic acid contents in oat genotypes as; 2.71 and 7.16%, 23.63 and 41.38%, 33.66 and 52.99%, 17.50 and 20.93%, 1.37, and 2.00% respectively.

Appreciable variations in moisture (7.81–11.95%), ash (3.88–8.89%), and crude fiber contents (10.27–19.67%) were recorded as well. The present investigation is in agreement with that of the reported study of Youssef et al., 2016, where the authors have observed a significant variation in crude protein, moisture content, ash content, and crude fats. Kose et al., (2021) reported the distribution of the proximate component as; ash content in the range of 2.10–2.25%, starch content in the range of 43.0–45.0%, β-glucan content in the range of 2.4–2.7%. Usman et al., (2010) reported the ash content in oat genotypes which were ranged from 1.87 to 4.33%. Such variations are encountered as the developmental conditions, genetic factors, environmental factors, regional variations, and analytical approaches influence the results (Alam et al., 2020). Other factors like harvesting conditions, storage, and post-harvest treatments can also lead to variations in the proximate components (Dhanalakshmi, 2021).

In developing countries, grain, which is a good source of minerals, is used as a staple food. Minerals are necessary elements in human nutrition. The human body cannot produce minerals, so minerals must be supplied through food (Biel et al., 2020). A considerable variation was observed among the germplasms for the studied elements; magnesium (2.89–7.62 mg/L), sodium (3.71–8.03 mg/L), and manganese (0.93–3.71 mg/L). The minerals are important for proper growth of animal and human which plants concentrates in their tissues through phytoremediation. Mut et al., (2017) reported oat genotypes to contain 4.99%, 3.66%, and 1.50% of K, P, and Mg contents, respectively. According to Kose et al., (2021) K, P, and Mg contents in oats are in ranges 2.78–6.73 gkg^−1^, 3.44–5.47 gkg^−1^, and 1.16–1.90 gkg^−1^. Ciolek et al., 2007, Gambuś et al., 2011 have also observed significant variations in oat germplasms for K, P, Mg Ca, Mn, and Cu. The variations for other studied metals were as: Zn; 1.30–3.37 mg/L, copper; 0.35–3.36 mg/L, and iron; 2.15–6.82 mg/L. The study of De-Oliveira et al., (2021) showed Zn concentration was from 27.3 and 57.7 mgkg^−1^, Fe from 38 to 63 mgkg^−1^, Mn from 59 to 105 mgkg^−1^_,_ Cu from 4.1 to 7.6 mgkg^−1^ in the grain of white oat genotypes. The Zn accumulation found in this study is similar to that of other grains like wheat, maize, and rice (Velu et al., 2019). The zinc concentration in the rye (16–24 mg kg^−1^) and barley (6–33 mg kg^−1^).

To correlate the compositional variations in plants Principal Component Analysis is used. The PC1, PC2, PC3, and PC4 depicted the total variation to be 85.01% while in the case of elemental analysis the total variation was 73.75%. Accession 769 and 817 were found to be the most diverse germplasm as depicted by nutritional component PCA while based on elemental analysis the accession 769 and 22,350 were the most diverse lines. Our results are in line with the reported study of Bhardwaj et al., (2019) where the authors have studied 43 oat germplasms. Kose, Mut, & Akay, (2021) screened 347 oat genotypes in the year 2012–2014 where PCA outcome indicated that 84% of the total variability among the genotypes.

Correlation analysis is important among the different nutritional contents as for a cereal it is important to have a proportionate composition. Negative and positive correlations were observed among different components as per giver details: crude protein and carbohydrate, negative; ash content with carbohydrate, and crude protein, positive; moisture content and crude protein, positive; crude fibers with crude protein and ash content, also positive. The recorded observations were in line with the reported study literature (Bhardwaj et al., 2019).

Consumption of oat has been associated with various health benefits that may be attributed to their nutritional value and phytochemical composition. Phytochemicals are the secondary metabolites that act as part of the defense system against diseases in plants. A total of nine phenolic compounds as given in the result section viz; malic acid, epigallocatechin gallate, quercetin, morin, ellagic acid, catechin hydrate, rutin, pyrogallol, mandelic acid have been identified in various oat germplasm. The compounds mentioned are associated with several health advantages if consumed by humans as reported by Nazir et al., 2018, Nazir et al., 2020. Most of these compounds are antioxidants, thus the use of oats would have substantial health benefits if consumed as cereal by humans. Although the present consumption of oats is lower than other cereals across the globe, it is getting popular in scientific and common communities due to its potential health benefits. As a source of antioxidants, it has been reported by other researchers as well and according to their findings, various phenolic compounds (phenolic acids, flavonoids, stilbenes, coumarins, and tannins) and phytic acid (Gangopadhyay et al., 2015) are responsible for the mentioned biological potential. In addition to their dietary importance, oat antioxidants may also contribute to the stability and the taste of food products by donating their hydrogen to free radicals and prevent autoxidation (Redaelli et al., 2016).

The storage protein in oats has been found to have the presence of bioactive peptides which may play an important role in the prevention and control of various chronic diseases such as diabetes, cancer, and age-related issues, along with the advantage of having fewer side effects compared to the synthetic counterparts (Guo et al., 2014). Oats β-glucan lower glycemic index and postprandial glucose levels (Gupta and Mishra 2021). Avenanthramides are another class of polyphenols that have been reported from oat which act as an antioxidant by inhibiting low-density lipoprotein oxidation with the aid of Vitamin C (Pellegrini et al., 2016). It has also shown numerous other beneficial health-promoting properties such as antiproliferative, anti-itch, anti-inflammatory, anticancer, and antiatherogenic effects and thus may be useful in the prevention of skin irritation, colon cancer, and coronary heart diseases. Bioactive compounds like coumarins, saponins, flavonoids, flavones, proteins, tocols, β-glucan, and carbohydrates have also been reported from this plant by Singh et al. (2019).

## 5 Conclusions

Cereals are grown for their nutritionally edible grains and are consumed all over the world. Oats are the world's sixth most important grain crop, following wheat, corn, rice, barley, and sorghum. Oats have the potential to be a competitor in the category of super-food because they offer a wide range of health-promoting characteristics and nutritional benefits. It contains all principle nutritional components like carbohydrates, protein, vitamins, minerals, antioxidants, and soluble fiber. Between 2018 and 2019, 236 oat genotypes were tested for agro morphological and biochemical characterizations at the Botanical Garden University of Malakand, Khyber Pakhtunkhwa, Pakistan. 30 potential high-yielding seeds were subjected to proximate and elemental composition analyses to discover the most effective variant. The present study has not only confirmed the presence of these ingredients but also has depicted variation in their quantities among different germplasms which would enable the breeder to select the best varieties for future cultivars. Because of their nutritional value and health benefits, oats have become increasingly popular. The accessions; 725, 22365, 22348, 830, 22350, 841, and 832 were found to have high nutritional components like carbohydrate, proteins, ash content, fats, and fibers. However, further confirmatory studies are needed to confirm the observed parameters in other localities too as the plant production, minerals, and phytochemical compositions vary with locality and particularly with the type of soil.

## Declaration of Competing Interest

The authors declare that they have no known competing financial interests or personal relationships that could have appeared to influence the work reported in this paper.
