# Supplementary material for: Genetic diversity in nutritional composition of oat (Avena sativa L.) germplasm reported from Pakistan
Source: Saudi J Biol Sci. 2021 Nov 19;29(3):1487–500. doi: 10.1016/j.sjbs.2021.11.023 (PMC8913558; doi:10.1016/j.sjbs.2021.11.023)
Supplement: Supplementary data 1 [file mmc1.docx]

**Supplementary file**

**Tables and Figures**

**Table S1** Principle component analysis of different parameters of oat using proximate analysis.

| **AXIS** | **PC1** | **PC2** | **PC3** | **PC4** |
| --- | --- | --- | --- | --- |
| Carbohydrate | -0.46 | 0.56 | -0.05 | 0.15 |
| Dry Matter | 0.04 | 0.23 | 0.35 | -0.88 |
| Protein | 0.53 | 0.26 | -0.11 | -0.12 |
| Ash content | 0.45 | -0.33 | -0.42 | -0.11 |
| Moisture content | -0.36 | -0.34 | -0.38 | -0.26 |
| Fats | 0.37 | 0.08 | 0.47 | 0.34 |
| Fibers | -0.18 | -0.58 | 0.57 | 0.00 |
| Cum. % of Var. | 33.78 | 54.75 | 71.02 | 85.01 |
| Eigenvalue | 2.59 | 1.59 | 1.09 | 0.76 |

**Table S2** Mean performance of different parameters (carbohydrate, dry matter, ash content, crude protein, moisture content, crude Fats, and crude fiber) among clusters.

| **Parameters**  **Mean±SD** | **Cluster 1** | **Cluster 2** | **Cluster 3** | **Cluster 4** | **Cluster 5** |
| --- | --- | --- | --- | --- | --- |
| Carbohydrates | 48.11±1.61 | 53.10±0.30 | 45.37±1.06 | 49.45±0.91 | 38.20±0.98 |
| Dry Matter | 90.66±1.20 | 91.32±1.99 | 91.01±1.80 | 91.53±2.05 | 90.09±2.00 |
| Protein | 15.81±0.81 | 13.33±0.80 | 14.38±1.16 | 11.29±0.66 | 16.01±2.39 |
| Ash Content | 5.93±1.24 | 6.08±1.47 | 5.38±1.62 | 4.85±1.05 | 8.39±0.70 |
| Moisture content | 9.35±1.00 | 9.39±0.76 | 9.27±1.00 | 10.79±1.57 | 9.25±1.56 |
| Fats | 6.35±0.85 | 6.29±0.72 | 6.80±0.50 | 5.57±0.70 | 6.93±1.03 |
| Fibers | 14.71±1.88 | 11.85±2.03 | 18.81±0.04 | 18.04±1.33 | 16.08±0.09 |

**Table S3** Different parameters of oat using elemental analysis, Eigenvalues, percentage of cumulative variability that contributed towards principal components.

| **AXIS** | **PC1** | **PC2** | **PC3** | **PC4** |
| --- | --- | --- | --- | --- |
| Magnesium | 0.04 | -0.01 | 0.68 | 0.09 |
| Sodium | 0.42 | 0.20 | -0.41 | 0.24 |
| Manganese | 0.34 | 0.37 | -0.17 | -0.48 |
| Potassium | 0.10 | -0.43 | 0.21 | -0.61 |
| Chromium | 0.54 | -0.38 | 0.13 | 0.06 |
| Zinc | -0.17 | 0.57 | 0.22 | -0.40 |
| Copper | 0.17 | 0.36 | 0.45 | 0.38 |
| Iron | 0.57 | 0.13 | 0.11 | -0.08 |
| Eigenvalue | 1.71 | 1.70 | 1.51 | 0.97 |
| Cum. % of Var. | 21.42 | 42.70 | 61.62 | 73.75 |

**Table S4** Mean performance of different parameters (Magnesium, Sodium, Manganese, Potassium, Chromium, Zinc, Copper, and Iron) among clusters.

| **Parameters Mean±SD** | **Cluster 1** | **Cluster 2** | **Cluster 3** | **Cluster 4** | **Cluster 5** | **Cluster 6** | **Cluster 7** |
| --- | --- | --- | --- | --- | --- | --- | --- |
| Magnesium | 5.03±1.45 | 4.55±0.34 | 6.28±1.04 | 6.72±0.59 | 3.17±0.24 | 5.12±0.66 | 6.75±1.19 |
| Sodium | 5.54±1.63 | 6.87±0.13 | 5.93±0.66 | 4.10±0.38 | 6.49±0.95 | 6.51±0.74 | 8.03±0.01 |
| Manganese | 1.56±0.68 | 2.50±0.76 | 1.93±0.62 | 2.15±0.10 | 2.19±0.34 | 1.41±0.29 | 2.97±1.04 |
| Potassium | 53.87±0.40 | 51.31±0.61 | 53.20±1.00 | 57.31±2.04 | 54.26±1.40 | 56.25±1.33 | 54.88±1.49 |
| Chromium | 0.91±0.53 | 1.05±0.67 | 1.59±0.78 | 1.61±0.98 | 2.37±0.27 | 1.84±0.80 | 2.62±0.35 |
| Zinc | 1.91±0.40 | 2.61±0.63 | 2.43±0.55 | 2.65±0.85 | 1.59±0.33 | 1.99±0.18 | 2.17±0.21 |
| Copper | 0.91±0.71 | 2.67±0.47 | 2.45±0.64 | 1.90±0.93 | 1.13±0.69 | 2.05±0.34 | 1.31±0.28 |
| Iron | 2.90±0.81 | 2.89±1.27 | 5.41±1.06 | 3.61±0.69 | 5.32±0.86 | 5.23±0.53 | 6.55±0.05 |

**
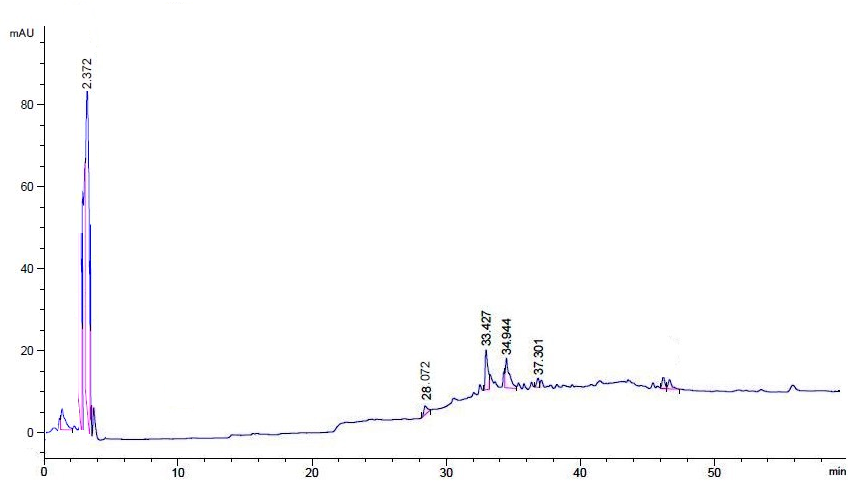
**

**Fig. S1.** HPLC chromatogram of oat germplasm-832.

**
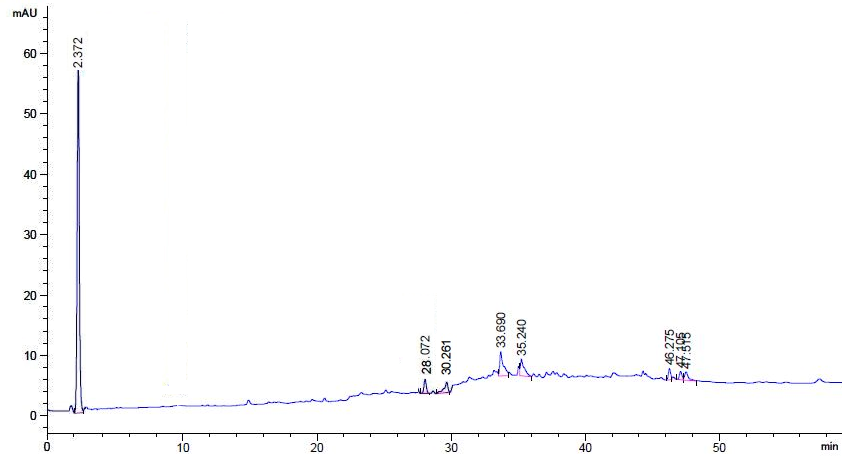
**

**Fig. S2.** HPLC chromatogram of oat germplasm-791

**
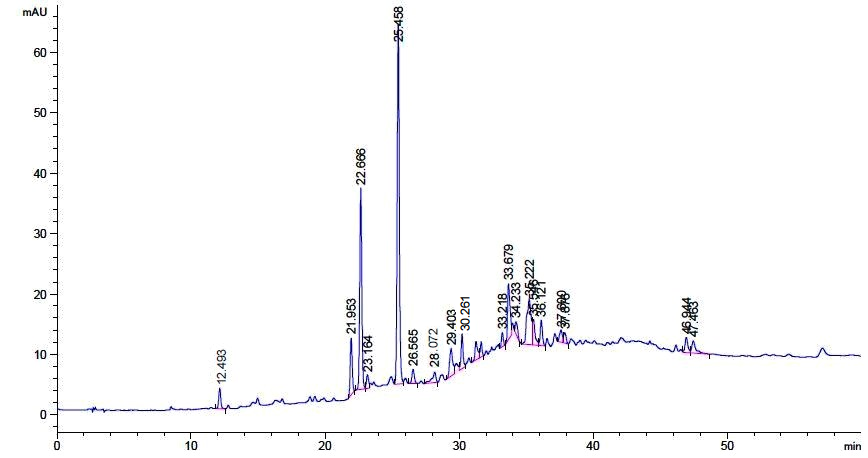
**

**Fig. S3.** HPLC chromatogram of oat germplasm-672


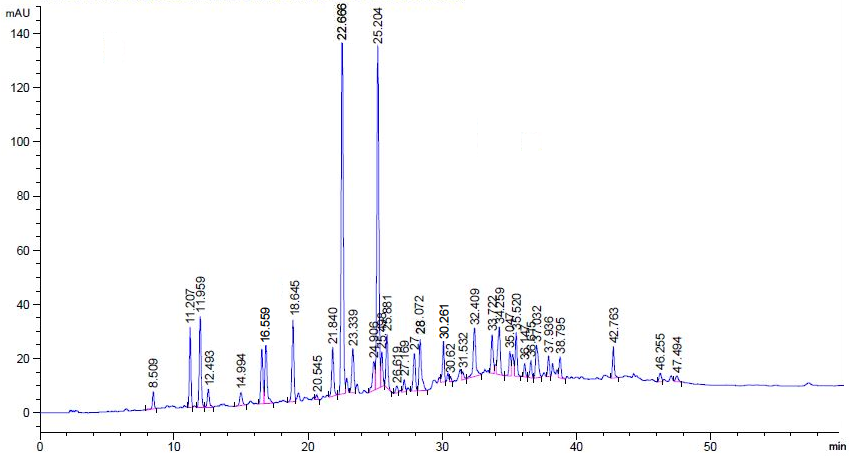


**Fig. S4.** HPLC chromatogram of oat germplasm-22348

**
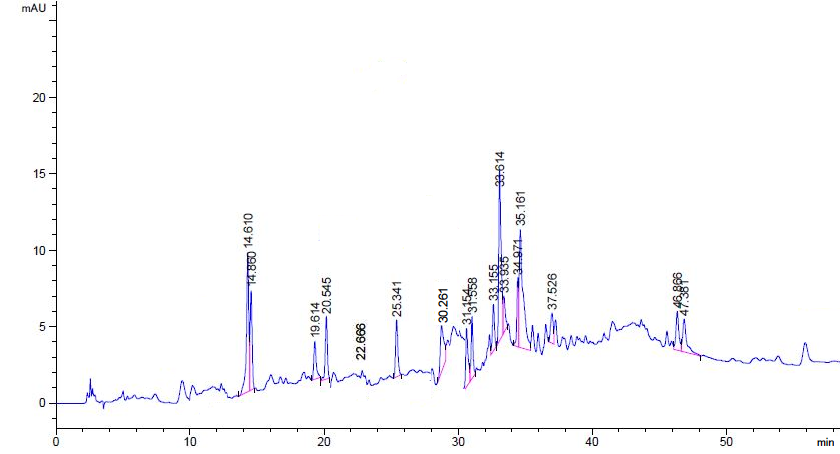
**

**Fig. S5.** HPLC chromatogram of oat germplasm- 726

**
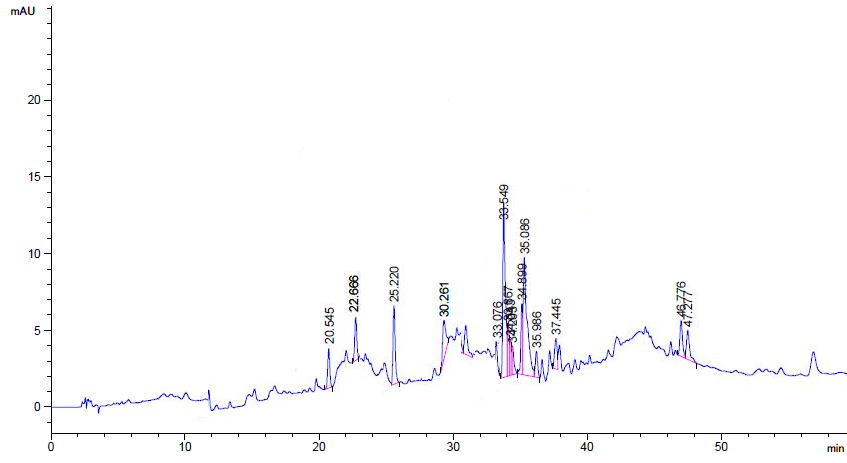
**

**Fig. S6.** HPLC chromatogram of oat germplasm- 22393

**
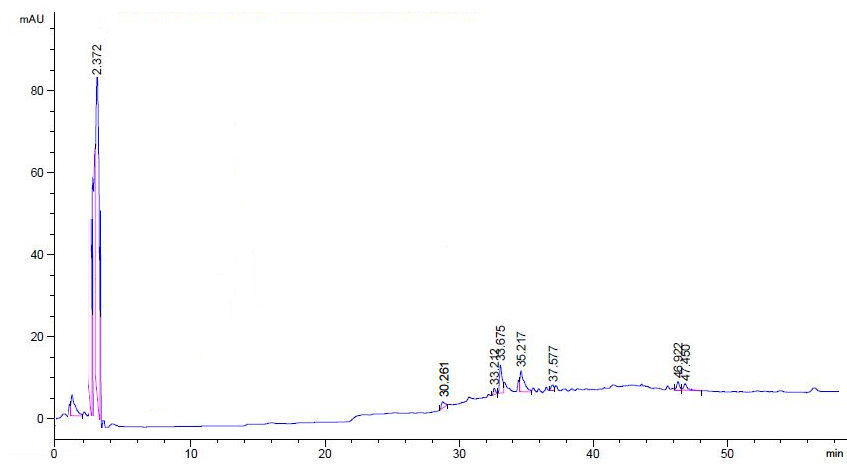
**

**Fig. S7.** HPLC chromatogram of oat germplasm-839.

**
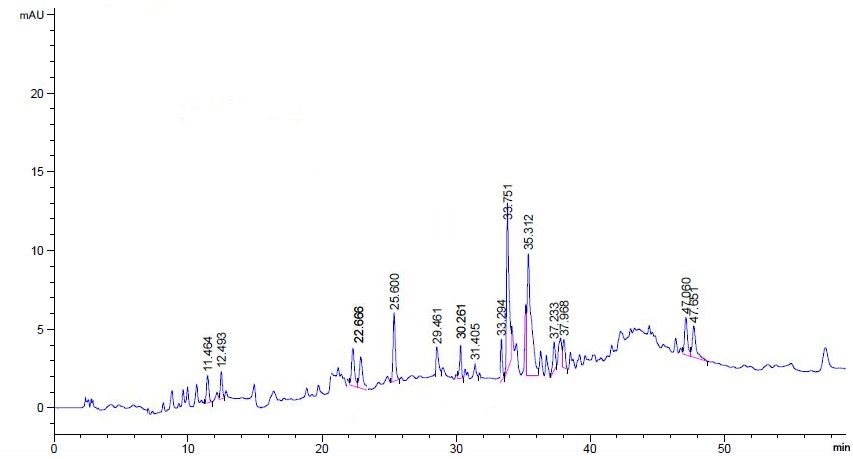
**

**Fig. S8.** HPLC chromatogram of oat germplasm-22347

**
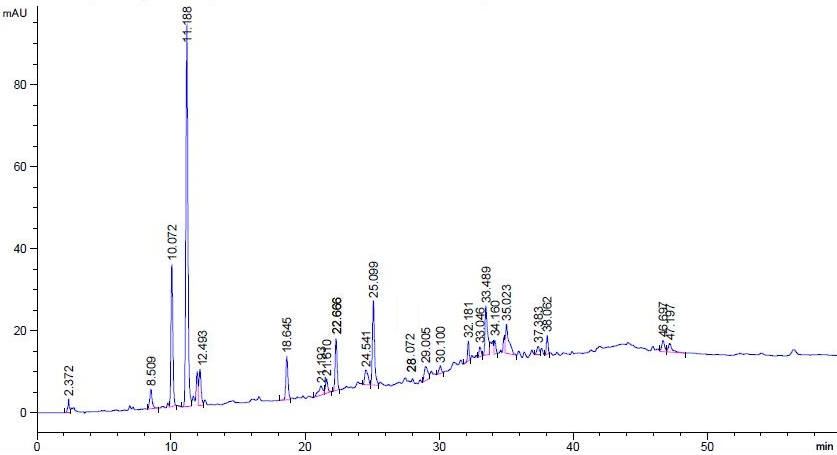
**

**Fig. S9.** HPLC chromatogram of oat germplasm-811

**
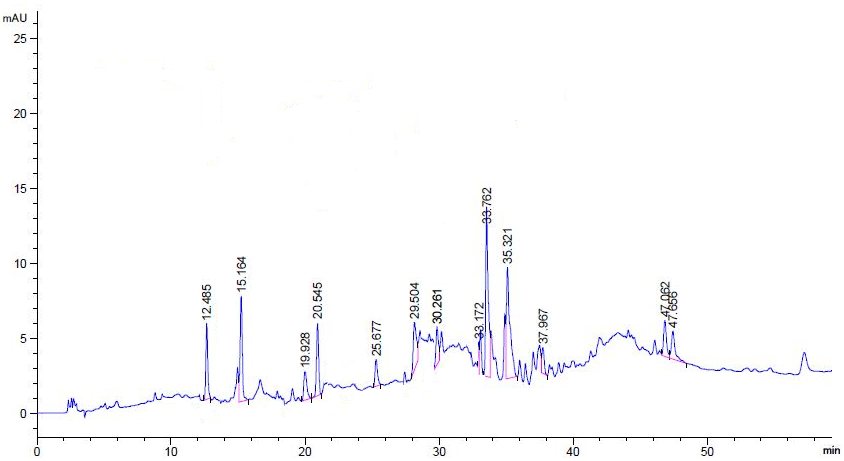
**

**Fig. S10.** HPLC chromatogram of oat germplasm- 830

**
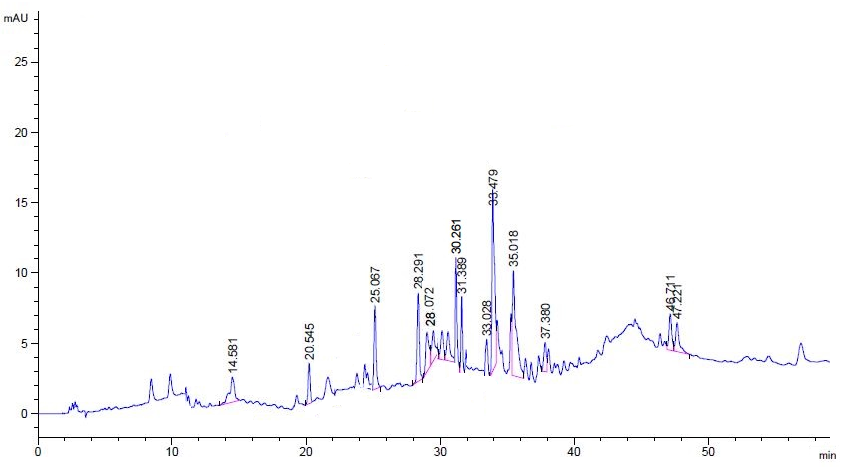
**

**Fig. S11.** HPLC chromatogram of oat germplasm-841

**
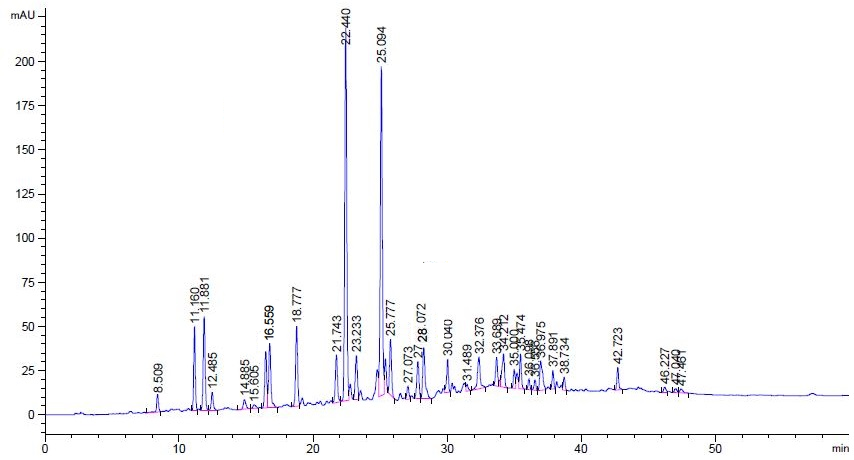
**

**Fig. S12.** HPLC chromatogram of oat germplasm- 843

**
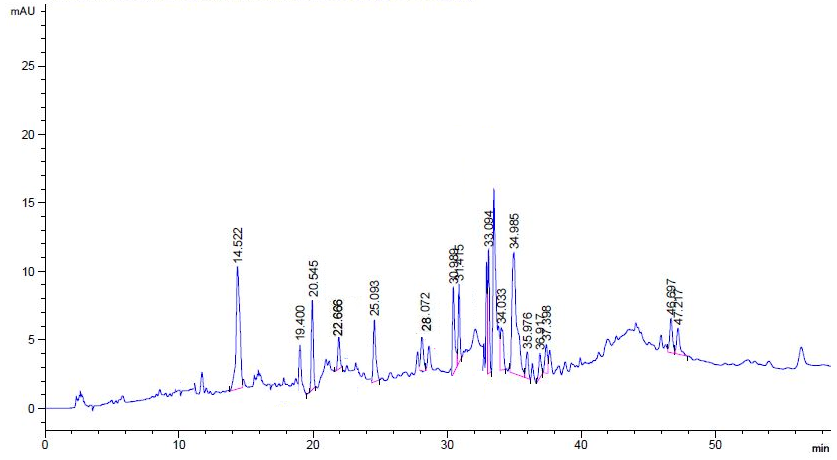
**

**Fig. S13.** HPLC chromatogram of oat germplasm- 22390

**
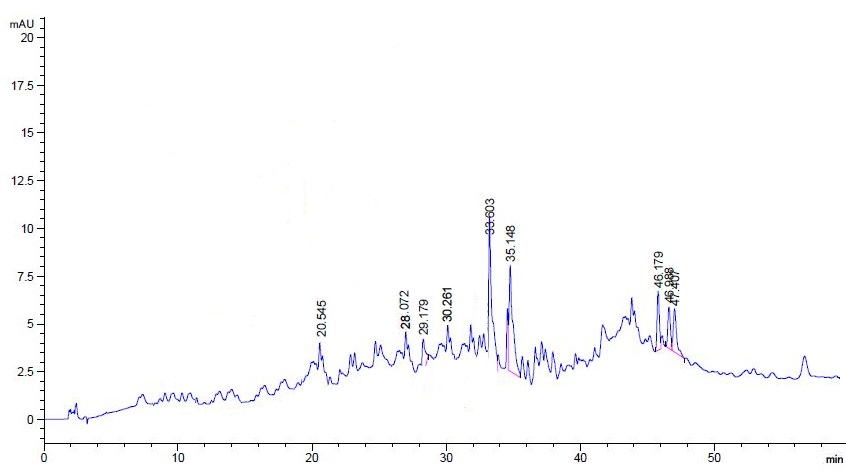
**

**Fig. S14.** HPLC chromatogram of oat germplasm- 22351

**
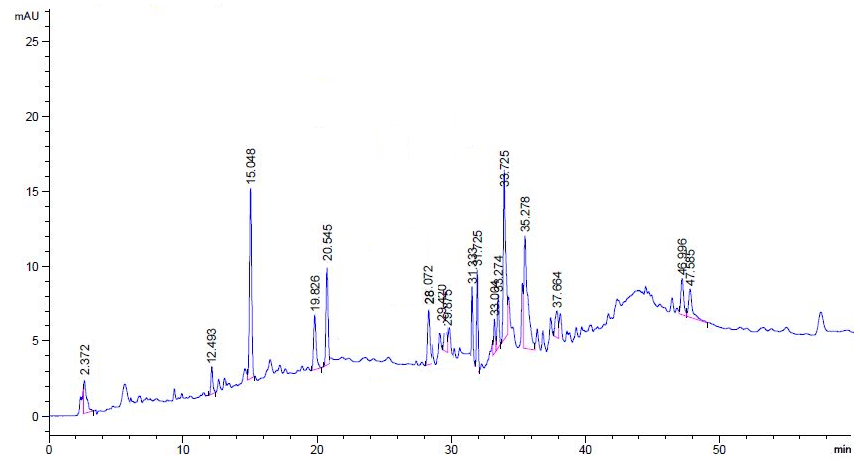
**

**Fig. S15.** HPLC chromatogram of oat germplasm- 22335
